# Clinical and Genetic Profiles of 5q- and Non-5q-Spinal Muscular Atrophy Diseases in Pediatric Patients

**DOI:** 10.3390/genes15101294

**Published:** 2024-09-30

**Authors:** Hisahide Nishio, Emma Tabe Eko Niba, Toshio Saito, Kentaro Okamoto, Tomoko Lee, Yasuhiro Takeshima, Hiroyuki Awano, Poh-San Lai

**Affiliations:** 1 Faculty of Rehabilitation, Kobe Gakuin University, 518 Arise, Ikawadani-cho, Nishi-ku, Kobe 651-2180, Japan; 2 Laboratory of Molecular and Biochemical Research, Biomedical Research Core Facilities, Graduate School of Medicine, Juntendo University, 2-1-1 Hongo, Bunkyo-ku, Tokyo 113-8421, Japan; niba@juntendo.ac.jp; 3 Department of Neurology, National Hospital Organization Osaka Toneyama Medical Center, 5-1-1 Toneyama, Toyonaka 560-8552, Japan; saito.toshio.cq@mail.hosp.go.jp; 4 Department of Pediatrics, Ehime Prefectural Imabari Hospital, 4-5-5 Ishi-cho, Imabari 794-0006, Japan; kentaro206@gmail.com; 5 Department of Pediatrics, Hyogo Medical University, 1-1 Mukogawacho, Nishinomiya 663-8501, Japan; to-ri@hyo-med.ac.jp (T.L.); ytake@hyo-med.ac.jp (Y.T.); 6 Organization for Research Initiative and Promotion, Research Initiative Center, Tottori University, 86 Nishi-cho, Yonago 683-8503, Japan; awano@tottori-u.ac.jp; 7 Department of Pediatrics, National University of Singapore, 1E Lower Kent Ridge Road, Singapore 119228, Singapore; paelaips@nus.edu.sg

**Keywords:** 5q-SMA, non-5q-SMA, SMARD1, SMA-JI, XL-SMA, SP-SMA, SMA-PME, SMA-LED1, SMA-LED2

## Abstract

Background: Spinal muscular atrophy (SMA) is a genetic disease characterized by loss of motor neurons in the spinal cord and lower brainstem. The term “SMA” usually refers to the most common form, 5q-SMA, which is caused by biallelic mutations in *SMN1* (located on chromosome 5q13). However, long before the discovery of *SMN1*, it was known that other forms of SMA existed. Therefore, SMA is currently divided into two groups: 5q-SMA and non-5q-SMA. This is a simple and practical classification, and therapeutic drugs have only been developed for 5q-SMA (nusinersen, onasemnogene abeparvovec, risdiplam) and not for non-5q-SMA disease. Methods: We conducted a non-systematic critical review to identify the characteristics of each SMA disease. Results: Many of the non-5q-SMA diseases have similar symptoms, making DNA analysis of patients essential for accurate diagnosis. Currently, genetic analysis technology using next-generation sequencers is rapidly advancing, opening up the possibility of elucidating the pathology and treating non-5q-SMA. Conclusion: Based on accurate diagnosis and a deeper understanding of the pathology of each disease, treatments for non-5q-SMA diseases may be developed in the near future.

## 1. Introduction

Spinal muscular atrophy (SMA) is an inherited disorder characterized by motor neuron loss in the spinal cord and lower brainstem [1]. In 1995, the *Survival Motor Neuron 1* gene (*SMN1*) mapped on chromosome 5q13 was identified as the causative gene of the most common form of SMA disease [2]. Since then, this form has been called 5q-SMA (in fact, 5q-SMA is now simply referred to as “SMA”), and DNA-based testing by polymerase chain reaction (PCR) has been widely used for its diagnosis.

At the beginning of the 21st century, three treatments for 5q-SMA were developed: nusinersen, onasemnogene abeparvovec, and risdiplam [3]. Clinical studies have shown that treatment for 5q-SMA is more effective the earlier it is started. Therefore, newborn screening (NBS) programs have been implemented worldwide to enable the early diagnosis of this disease [4]. However, non-5q-SMA still remains incurable. The terms “5q-SMA” and “non-5q-SMA” are now commonly used to refer to curable and incurable SMA, respectively, making this a simple and practical classification.

Awareness of non-5q-SMA among the general public remains inadequate. When a newborn baby is diagnosed with 5q-SMA by NBS, the parents may be shocked and then worried about choosing the best treatment [5]. However, the parents of babies diagnosed with non-5q-SMA face much greater challenges given that non-5q-SMA is even less well-known. The parents may believe that SMA can be ruled out because the NBS result for 5q-SMA was negative. A subsequent diagnosis of non-5q-SMA, which cannot be cured with current drugs, would be devastating. Indeed, a family experiencing such a situation has already been encountered in Japan: one family reported on social media that they had given birth to a baby with non-5q-SMA, who tested negative for prior NBS of 5q-SMA. In this context, healthcare providers, including pediatricians, should be knowledgeable enough to explain 5q-SMA and non-5q-SMA diseases to parents. (To be more precise, we must avoid the term “cure” for 5q-SMA. Although promising therapy options are available, it is too early to talk about a “cure” for 5q-SMA.)

When research on non-5q-SMA disorders was in its infancy, investigators mainly focused on classifying the condition based on its clinical features. Later, focus shifted toward identifying causative genes of the different non-5q-SMA disease types. Many genes have been discovered through studying pedigrees, combined with linkage analyses [6]. Diagnosis of non-5q-SMA cases at the DNA level is now possible by using next-generation sequencing (NGS) technologies such as whole-exome sequencing (WES) and whole-genome sequencing (WGS). Clinical and basic knowledge of non-5q-SMA diseases has been accumulating, providing a deeper understanding of their pathogenesis [7,8]. A new era of research is now underway, in which novel therapeutic strategies, including gene therapy [9], are being developed for non-5q-SMA diseases. (A few caveats should be added here: NGS techniques (WES/WGS) are the methods of choice, but the diagnostic yield is still low. It is expected that these new techniques will be further improved and become more widely used in clinical practice.)

In this review article, we present a non-systematic critical review summarizing the clinical and genetic profiles of 5q-SMA and non-5q-SMA diseases in pediatric patients. Although many non-5q-SMA types are recognized as being adult-onset and slowly progressive disorders, others are known to be acute conditions that first manifest in infancy and childhood. We hope that his overview of 5q-SMA and representative non-5q-SMA diseases in pediatric patients will be useful for pediatric healthcare providers including pediatricians.

## 2. Classification of SMA

### 2.1. SMA Cases with Different Clinical Phenotypes and Genetic Backgrounds

In 1891, Werdnig reported the first two cases of SMA with a phenotype similar to SMA type II (current classification) [10]. Since then, many SMA cases with different clinical phenotypes and genetic backgrounds have been reported. SMA was classified on the basis of the clinical phenotype, age of onset, pattern of muscle involvement, and mode of inheritance. The clinical and genetic heterogeneity of SMA led to divided opinions on whether to continue subdividing SMA patients into distinct subgroups, or to regard the patients as part of a broader spectrum [11].

Some researchers suggested that SMA presents a continuous spectrum from severe through intermediate to mild phenotypes and proposed that these different phenotypes can be attributed to a single genetic disorder with variable clinical expression [12]. However, other researchers were concerned that this concept of a continuous spectrum could lead to genetically heterogeneous neurological diseases being misclassified into a single category [13].

### 2.2. Genetic Homogeneity of the Most Common Form of SMA, 5q-SMA

In 1990, Gilliam et al. in the US and Melki et al. in France mapped the gene locus of chronic SMA (intermediate and mild SMA) and acute SMA (severe SMA) to the same region of chromosome 5q. All of these studies used linkage analyses. According to the report by Gilliam et al., the locus associated with SMA was identified at chromosome 5q11.2–13.3 [14,15], while Melki et al. localized it to chromosome 5q12–14 [16,17].

In 1995, Lefebvre et al. identified the causative gene, *SMN1*, in the SMA locus of chromosome 5q13 [2]. Since then, the most common, proximal form of SMA has been called SMN1-related SMA or 5q-SMA. The identification of *SMN1* as a 5q-SMA-causing gene accelerated progress in the diagnosis and treatment of this disease. The ability to diagnose 5q-SMA at the molecular level has enabled more accurate epidemiological study. Indeed, 5q-SMA is now known to occur in one in 10,000–20,000 live births all over the world [18], and more than 95% of 5q-SMA patients are homozygous for *SMN1* deletion [2,19,20].

At the beginning of the 21st century, three new drugs for 5q-SMA were released in rapid succession: nusinersen, onasemnogene abeparvovec, and risdiplam [3].

After genetic homogeneity of the most common, proximal form of SMA was proven in 1990, and a 5q-SMA-causing gene was identified in 1995, the idea of a continuous spectrum of SMA became accepted by physicians worldwide. Reflecting this, 5q-SMA is currently simply referred to as “SMA”. However, the category of SMA actually includes genetically heterogeneous neurological diseases. In fact, long before *SMN1* was discovered, researchers had already reported the existence of non-5q-SMAs including the distal form of SMA (distal SMA) [11].

### 2.3. Genetic Heterogeneity of Non-5q-SMA

In 1980, Pearn created a classification table of SMA that included non-5q-SMA (of course, in Pearn’s terminology, neither “5q-SMA” nor “non-5q-SMA” was used) [11]. In this classification system, the different SMA syndromes were discriminated by (a) age at the first clinical signs of the disease, (b) pattern of muscle involvement, (c) age at death of other patients within the affected kindred, and (d) genetic evidence.

On the basis of an English SMA study, Pearn defined seven SMA classes, which were clinically and genetically different [11]. According to Pearn’s report, SMA type I (Werdnig–Hoffman disease) and chronic childhood SMA together account for 74% of all SMA patients. By considering Pearn’s report based on our current knowledge of 5q-SMA, SMA type I and chronic childhood SMA in Pearn’s classification could together be classified into 5q-SMA types I, II, and III of our current classification [1,21]. Thus, roughly speaking, 74% of the patients would be classified with 5q-SMA, and the rest (26%) with non-5q-SMA.

However, in 2018, Karakaya et al. reported a genetic study with a large cohort of 3465 individuals in Europe and the Middle East suspected of having SMA who underwent *SMN1* testing as part of their routine diagnostic laboratory work-up [22]. According to that study, 48.8% of the participants were homozygous for *SMN1* deletion, 2.8% were compound heterozygous for a subtle mutation and an *SMN1* deletion (thus 51.6% were affected by 5q-SMA), whereas 48.4% remained undiagnosed. Then, the clinical utility of targeted sequencing in non-5q-SMA was further tested by developing two different gene panels, which identified causative gene mutations in many, but not all, participants [22]. Table 1 summarizes the similarities and differences between 5q-SMA and non-5q-SMA. The data of Karakaya et al. are presented in adapted form in this table.

We also have similar data to those of Karakaya et al. We analyzed a total of 515 Japanese patients with SMA-like symptoms (delayed developmental milestones, respiratory failures, muscle weakness, etc.) from 1996 to 2019 [23]. A total of 241 patients were diagnosed as having 5q-SMA. However, we could not identify the gene abnormality in the 274 patients because the new technologies were not available in our laboratory. Having had such experiences, we could easily accept the data of Karakaya et al. The statistics in Table 1 come from a few routine diagnostic laboratories where the detailed clinical information might not always be accurate. Further research is required to determine the global incidence of non-5q-SMA.

### 2.4. Charcot–Marie–Tooth Diseases and Hereditary Motor Neuropathies

Pearn mentioned “Distal SMA” in his classification table [11]. He also presented in the table the term “Progressive SMA—Charcot–Marie–Tooth disease (CMT) type” as the principal synonym of “Distal SMA”. SMA has been understood as central nerve diseases in the spinal cord, with CMT as peripheral nerve diseases. However, the peripheral nerves diverge from the central nervous system, which suggests the disease continuity between them. Pearn may have implied in the table that there is overlap between SMA and CMT, or that the boundaries between them are blurred.

CMT was first described 120 years ago as a familial neurological syndrome called “peroneal muscular atrophy”. [24]. CMT is now recognized as the most common form of genetic neuropathy, which affects both motor and sensory peripheral nervous systems. CMT is electrophysiologically divided into demyelinating [CMT1; upper limb motor conduction velocity (MCV) < 38 m/s] and axonal forms (CMT2; MCV ≥ 38 m/s) [25]. CMT1 is considered a demyelinating CMT, while CMT2 is considered an axonal CMT. SMA is thought to be similar to CMT2 diseases.

CMT is often referred to as hereditary motor and sensory neuropathy (HMSN). Recently, the terms “hereditary sensory neuropathy” (HSN) and “hereditary motor neuropathy” (HMN) have frequently been used. HSN and HMN refer to forms of CMT that affect sensory and motor nerves, respectively [25]. HSN with variable autonomic involvement can be grouped into “hereditary sensory and autonomic neuropathy” (HSAN) [26]. The HMNs with minimal to no sensory involvement and the HSNs with significant sensory involvement might be opposite ends of a continuous spectrum of CMT phenotypes [27].

Distal SMA is also referred to as distal HMN [28]. Thus, Farrer et al.’s classification combines distal SMA and distal HMN into a single category, distal SMA/HMN [1]. The distal SMA/HMNs are a genetically heterogeneous group of diseases characterized by distal lower motor neuron weakness, which often overlaps with the axonal form of CMT [28,29]. These conditions share features such as a slowly progressive, nerve-fiber-length-dependent, distally predominant pattern of motor neuropathy. The length-dependent pattern, in which the toes and soles are affected first and hands later [30], involves the farthest nerve endings in the feet being the site where the symptoms develop first or are most severe. Significant sensory involvement is absent.

Non-5q-SMA, CMT2, and HMN diseases have been studied independently, but as the causative genes have been identified, it has become clear that mutations in the same genes cause non-5q-SMA, CMT2, and HMN diseases [31,32]. In other words, some genes that cause motor impairment have a history of being studied independently as non-5q-SMA-, CMT2-, and HMN-causative genes. To provide a historical and clinical context for non-5q-SMA, CMT2, and HMN, the terminology used in this review article is summarized in Table 2.

### 2.5. Current Classification of Non-5q-SMAs

In 2003, Zerres and Schöneborn published the results of a discussion on non-5q-SMA diseases at the 93rd ENMC International Workshop on Non-5q-Spinal Muscular Atrophies (SMA)—Clinical Picture (6–8 April 2001; Naarden, The Netherlands) [33]. In the workshop report, non-5q-SMA was discussed in two chapters: “SMA plus types” and “adult SMA”. In the “SMA plus types” chapter, diaphragmatic SMA (SMA with respiratory distress/SMARD) was described together with the *IGHMPB2* gene. Meanwhile, the “adult SMA” chapter highlighted the difficulty in distinguishing between distal SMA and axonal CMT. This report is useful for understanding how non-5q-SMA was viewed during that era. SMARD is now widely known among neonatologists and pediatricians as a non-5q-SMA disease that should not be overlooked in neonatal or pediatric wards. SMARD is also known as distal hereditary motor neuropathy type 6 (dHMN6) [1].

In 2015, Farrar et al. created a new classification table based on clinical and genetic studies of non-5q-SMAs [1]. This classification table exhibited three major characteristics. First, SMA was broadly divided into proximal and distal forms and so, at the highest hierarchical level, this classification was based on clinical symptoms. Second, distal SMA and distal HMN were combined and organized based on the original classification by Harding [34]. Third, causative genes that had been identified up to 2015 were listed in the table, showing the diversity of disease causes. In this classification system, Farrar et al. may have prioritized disease names for its clinical features over gene names. However, a single patient often combines clinical features of both proximal and distal lesions.

In 2023, Fernández-Eulate et al. reported a retrospective study on 71 adult patients with non-5q-SMA from 10 neuromuscular referral centers in France and UK [8]. On the basis of the clinical features, the patients were classified into three groups: (1) 21 patients with P-SMA who had pure proximal motor weakness at first visit, (2) 35 patients with PD-SMA who had proximal and distal weakness but pure proximal involvement at disease onset or proximal-predominant weakness at first visit, and (3) 15 patients with SP-SMA who had scapuloperoneal weakness.

In the study of Fernández-Eulate et al., 32 out of the 71 patients (45.1%) were genetically diagnosed [8]. The most common genes in non-5q-SMA patients were *BICD2* (*n* = 9) and *DYNC1H1* (*n* = 7), associated with SMA-LED, and TRPV4 (*n* = 4), implicated in SP-SMA. These three genes are dominantly inherited; however, only 13 out of 20 patients had a family history of the disease showing clear autosomal recessive inheritance, and thus the rest were categorized as sporadic cases.

Notably, the clinical features did not always distinguish the causative gene, and the causative genes did not always show the peculiar phenotypes. According to the data of Fernández-Eulate et al., *TRPV4* mutations were found only in 3 out of 15 patients with SP-SMA, although it is widely known that the causative gene for SP-SMA is *TRPV4* [8]. *BICD2* mutations were also found in 3 out of 15 patients with SP-SMA, although it is widely known that *BICD2* mutations cause SMA-LED2 [8]. Against this background, it may be time to reclassify 5q-SMA and non-5q-SMA based on new findings about the clinical features associated with related gene abnormalities.

### 2.6. SMA Diseases in Pediatric Patients Covered in This Article

Pediatric SMA diseases are listed in Table 3. In this review article, we describe SMA diseases due to the mutations of *SMN1* [2], *IGHMBP2* [35], *GARS* [36], *UBA1* [37], *TRPV4* [38,39,40], *ASAH1* [41], *DYNC1H1* [42], and *BICD2* [43,44,45], but not SMA diseases due to the mutations of *SCO2* [46], *LAS1L* [47], *TRIP4* [48], *ASCC1* [48], and *SPTBN4* [49].

Here, the eight diseases are summarized in the next sections as follows: (1) disease name and its abbreviation; (2) summary of the disease with an accompanying schematic; (3) clinical features with early case studies; (4) genetic information with mode of inheritance, causative genes, and gene products (mRNA or protein); and (5) molecular pathogenesis with fundamental defects.

As mentioned above, the clinical features are helpful in contributing toward the diagnosis of patients’ diseases, but they cannot accurately predict the outcome of a genetic test.

## 3. 5q-Spinal Muscular Atrophy (5q-SMA) [Alternative Title: SMN1-Related Spinal Muscular Atrophy (SMN1-Related SMA)]

### 3.1. Summary of the Disease

5q-SMA is a lower motor neuron disease with autosomal recessive inheritance. It is caused by biallelic disruption of *SMN1* on chromosome 5q13, which produces the SMN protein (hereafter, referred to as SMN). The incidence of 5q-SMA is 1 in 10,000 to 20,000 live births, and the carrier frequency is 1 in 40 to 70 individuals.

Muscle weakness is usually symmetrical, more proximal than distal, with the legs being more affected than the arms. However, the diaphragm, extraocular muscles, and facial muscles are relatively preserved.

**Figure 1:**
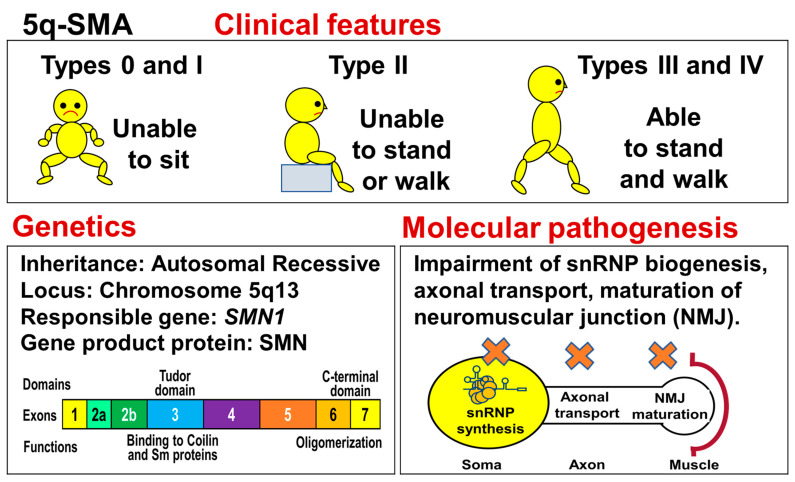
Outline of 5q-SMA. Patients with SMA types 0 and I are unable to sit, and often present with arthrogryposis (multiple joint contractures) and respiratory failure. Patients with SMA type II are able to sit, but unable to stand or walk, and almost always present with scoliosis. Patients with SMA type III are able to stand and walk, but they lose these abilities as the disease progresses. People with SMA Type IV can stand and walk, and because the disease progresses slowly, they can maintain these abilities for long periods of time. The protein encoded by the responsible gene has two main domains, Tudor domain and C-terminal domain [50]. The Tudor domain has binding sites to Sm proteins, which are components of snRNPs. The C-terminal domain contains a tyrosine/glycine-rich region (YG box) that plays a role in oligomerization of SMN. Oligomerization is an essential step for the various functions of SMN [51,52]. Defects in snRNP biogenesis, axonal transport and NMJ maturation are thought to be involved in the pathogenesis.

The disease usually begins in infancy or childhood and causes severe disability. There is a wide range of clinical severity, and the phenotype is divided into five types, Types 0 to IV. This classification is primarily based on maximum motor milestones achieved and age of onset. An outline of this disease is shown in Figure 1.

### 3.2. Clinical Features

As mentioned above, the incidence of 5q-SMA is 1 per 10,000 to 20,000 live births [18]. The frequency of carriers is thought to be 1 in 40 to 70 in the population [18]. Therefore, 5q-SMA can be said to be a relatively common “rare disease”.

Muscle weakness is usually symmetric, occurs more proximally than distally, and affects the legs more than the arms, although severe cases may have significant generalized weakness. Of note is the relative preservation of the diaphragm [12,53]. The diaphragm remains relatively intact and diaphragmatic breathing (paradoxical breathing) is possible [53]. Despite the diaphragm being relatively preserved, respiratory failure is a serious complication in severe cases of 5q-SMA [54].

5q-SMA is currently classified into five disease types (0, I, II, III, and IV), based on the age of onset and degree of achievement of motor milestones [1,21,50]. In this review article, we will adopt the current classification.

Type 0 is the severest type, which develops in the fetus. The affected infants exhibit respiratory difficulties from birth, and if respiratory care is not provided, death can occur within a few weeks [55].

Type I is a severe type, which develops before 6 months of age. The affected children are unable to sit up on their own and are often placed on mechanical ventilation, without which they die by the age of 2 years. In patients with Type I, a bell-shaped chest and paradoxical breathing (inspiratory chest wall-descent with abdominal expansion) are observed, reflecting intercostal muscle weakness and diaphragmatic sparing [53], as mentioned above.

Type II is an intermediate type, which develops by 18 months of age. The affected children can still sit up on their own, but are unable to stand or walk without support.

Type III is a mild type, which manifests after 18 months of age. The affected children can stand and walk without support. However, the ability to stand and walk is often lost with disease progression. Type III patients are expected to have a normal lifespan.

Type IV is the mildest type, which develops in adulthood. The affected individuals can stand and walk without support. Patients with SMA type IV are also expected to have a normal lifespan.

An earlier onset of the disease and a lower level of achieved motor milestones are associated with more severe features in patients. However, the classification described above is based on the natural history of patients not treated with newly developed drugs [3,50]. If treated with new drugs early in life, patients may be able to walk independently despite being genetically predicted to follow an SMA type I course [56,57,58].

At the end of the classification based on the motor function, we must also mention another clinical classification system (non-sitters, sitters, walkers), which is suggested for a better therapy follow-up [59]. This classification does not consider the age at onset and is based only on the current state, so it does not necessarily coincide with the above classification of types 0, I, II, III, and IV.

Here, we briefly discuss arthrogryposis (multiple joint contractures) of 5q-SMA. Arthrogryposis usually refers to joint contractures in multiple parts of the body, involving the extremities, but can also include a restricted range of movement in the jaw, neck, and spine at birth [60]. It was discussed whether arthrogryposis also occurs in 5q-SMA.

According to the report on the International SMA Consortium Meeting (1992) [61], arthrogryposis is an exclusion criterion for SMA (the term “SMA” used here refers to 5q-SMA). This assertion was made because marked arthrogryposis was not considered to be a common feature in 5q-SMA, although minor contractures of some joints were thought to be common [55]. Jug-handle posture of the arms with flexion contractures in 5q-SMA (Type I) patients is an example of minor contractures [12].

However, patients with the severest form of 5q-SMA (Type 0), whose symptoms start in the fetal period, have been identified [55,62,63,64], and they may present with marked arthrogryposis. Hence, 5q-SMA is currently not ruled out by the presence of arthrogryposis.

### 3.3. Genetics

In 1995, *SMN1* and *Survival Motor Neuron 2* (*SMN2*) genes were cloned from chromosome 5q13 as 5q-SMA-related genes [2]. *SMN1* (or ^T^BCD541 in the 1995 report) is present on the telomeric part, while the homologous gene, *SMN2* (or ^C^BCD541 in the 1995 report), is on the centromeric part.

5q-SMA is caused by biallelic mutations of *SMN1*. Overall, 95% of 5q-SMA patients (regardless of disease type) show a complete absence of both alleles of *SMN1*, while the rest retain one copy of *SMN1* with an intragenic mutation [2].

Meanwhile, no cases of 5q-SMA patients with the complete absence of *SMN2* have been reported. Instead, many studies have proven that *SMN2* copy number is associated with the severity of 5q-SMA. *SMN2* is currently thought to be a modifier that determines the severity of SMA [19,20,65,66].

*SMN1* produces full-length mRNA, leading to the production of full-length SMN. In contrast, *SMN2* produces mainly mRNA lacking exon 7, but it also produces a very small amount of full-length mRNA. The total amount of full-length SMN generated from *SMN2* in SMA patients may determine the clinical severity of the condition.

### 3.4. Molecular Pathogenesis

Full-length SMN is ubiquitous in tissues and organs of the human body. Dreyfuss et al. initially demonstrated the subcellular function of SMN, which is associated with snRNP biogenesis and splicing of pre-mRNA [67,68,69].

Nonetheless, many researchers believed that SMN defect in motor neurons triggered their selective degeneration. Thus, they searched for specific functions of SMN involved in the cellular activities of motor neurons [70,71]. Soon after, it was shown that full-length SMN is involved in axonal transport in motor neurons and the maturation of neuromuscular junctions (NMJs) [72].

To date, SMN has been implicated in the function of NMJs, involving the chemical transmission of electrical impulses from nerves to muscles [73]. Another molecular mechanism for pathogenesis of 5q-SMA has been described elsewhere [50].

However, it has not been clear whether sensory neurons are involved in 5q-SMA. In 1997, Bingham et al. conducted autopsy analyses with SMA patients, and reported that, in addition to the loss of anterior horn cells, the patients had degeneration of central sensory neurons in Clarke’s column and the thalamus [63].

Later, electrophysiological abnormalities were observed not only in motor nerves but also in sensory nerves of patients with 5q-SMA type I [74,75]. Imlach et al. also suggested, based on a study of *Drosophila* SMN mutants, that SMN depletion would first disrupt the sensorimotor circuit and then disrupt motor function [76]. Bottai and Adami also suggested that sensory-motor circuit dysfunction can be the starting point of motor system deficits in the 5q-SMA model [77]. If peripheral sensory neurons are found to be involved in 5q-SMA, then 5q-SMA would share motor and sensory neuron impairment with CMT.

Recently, microglial activation in neurodegenerative disorders has attracted attention [78]. In 2020, Ando et al. demonstrated that microglial activation may be related to the pathogenesis of 5q-SMA [79]. According to them, SMN regulates oxidative stress and inflammatory response in microglia. In SMN-deficient cells (RAW264.7), they observed increased production of reactive oxygen species and subsequent antioxidant stress responses. When SMN expression was increased with antisense oligonucleotide in SMA model mice, they found phenotypic improvement and suppression of microglial activity. In 2022, Wang et al. indicated, citing the paper of Ando et al., that microglia-related signaling and mediators may be involved in the development of motor neuron diseases [80].

## 4. Spinal Muscular Atrophy with Respiratory Distress Type 1 (SMARD1) [Alternative Titles: Distal Hereditary Motor Neuropathy Type 6 (dHMN6)/Charcot–Marie–Tooth Type 2S (CMT2S)]

### 4.1. Summary of the Disease

Spinal muscular atrophy with respiratory distress type 1 (SMARD1) is an autosomal recessive SMA presenting with progressive distal muscle weakness and respiratory failure due to diaphragmatic paralysis in early infancy. SMARD1 is caused by homozygous or compound-heterozygous mutation of the *Immunoglobulin Helicase Mu-binding Protein 2* (*IGHMBP2*) gene on chromosome 11q13. SMARD1 has also been referred to as distal spinal muscular atrophy 1 (DSMA1) and is also known as distal hereditary motor neuropathy type 6 (dHMN6 or HMN6).

**Figure 2:**
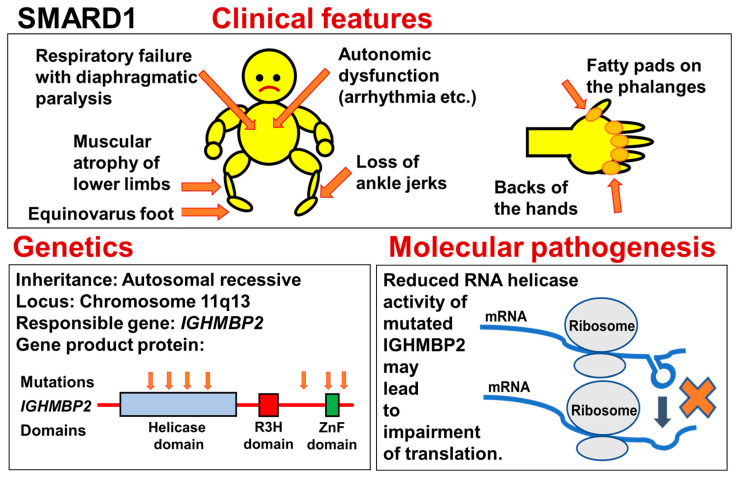
Outline of SMARD1. The illustration of clinical features was drawn based on a diagram provided by Perego et al. [81]. Causative mutations occur mainly in the helicase domain [82,83,84,85]. Mutations in the helicase domain of IGHMBP2 may lead to impairment of translation. However, the exact molecular mechanism of IGHMBP2-related disorders remains largely unknown. The illustration here focuses on the reduced RNA helicase activity of mutated *IGHMBP2*.

A second disorder due to *IGHMBP2* mutation shows a completely different phenotype. It is a disorder of the peripheral nervous system known as Charcot–Marie–Tooth type 2S (CMT2S). CMT2S is also an autosomal recessive disorder, but it does not bring about respiratory failure. Infantile SMARD1 and adult CMT2S could be considered opposite ends of the clinical spectrum of IGHMBP2-related disorders. An outline of SMARD1, is presented in Figure 2.

**Note:** Spinal muscular atrophy with respiratory distress type 2 (SMARD2) is an X-linked motor neuron disease with a similar phenotype to SMARD1 [47]. Patients with SMARD2 also present with diaphragm paralysis, distal muscular weakness, multiple joint contractures, and axial hypotonia. The phenotype overlaps considerably with SMARD1, but SMARD2 can be differentiated by the existence of a mutation in a different gene, the LAS1-like (*S. cerevisiae*) gene (LAS1L), by diagnostic exome sequencing. SMARD2 is also an infantile-onset, non-5q-SMA disease, but it will not be discussed here.

### 4.2. Clinical Features

In 1974, Mellins et al. reported two male infants with Werdnig–Hoffmann disease who presented with respiratory distress after an initial asymptomatic interval of 1 to 2 months [86]. Both patients were found to have bilateral diaphragmatic eventration (abnormal elevation) before loss of deep tendon reflexes or other signs of muscle weakness. The underlying progressive neuromuscular disease did not present until 3 to 4 months of age. Autopsy revealed widespread skeletal muscle atrophy and a thin diaphragm in these patients. Histological examination of various muscles revealed large groups of atrophic fibers, consistent with neurogenic changes.

In 1989, Bertini et al. reported five infants whose manifestations differed from recognized forms of SMA type I (Werdnig–Hoffmann disease) [87]. The hallmark features of these infants were congenital or early-onset diaphragmatic paralysis, muscle weakness, and atrophy, which were initially confined to the distal parts of the limbs. The muscle weakness and atrophy of the limbs progressed to include the proximal parts.

After a gene causative of diaphragmatic SMA, the *Immunoglobulin Mu* (*μ*) *Binding Protein 2* gene (*IGHMBP2*) was identified by Grohmann et al. in 2001 [35], various phenotypes associated with this gene’s abnormalities have come to be recognized. As a result, not only cases of SMARD1 in infancy but also those in childhood and beyond have been reported. For example, in 2004, Guenther et al. reported one patient with infantile-onset (4 months) and another with juvenile-onset (4.3 years) of respiratory distress [88]. These patients carried a compound-heterozygous mutation in *IGHMBP2.* Moreover, in 2015, Hamilton et al. reported a 21-year-old female patient with SMARD1 (juvenile SMARD1) whose diagnosis was genetically confirmed [85]. She had stable muscle weakness, and normal cognitive abilities, and was able to lead a socially integrated life, using mechanical ventilation only at night when sleeping.

*IGHMBP2* mutations were also found in CMT2 patients. In 2014, Cottenie et al. reported a total of 11 CMT2-affected families with recessively inherited *IGHMBP2* gene mutations [84]. Their first case involved an English family with two middle-aged sisters who had clinical features consistent with recessive CMT2 disease. Genetic analysis revealed compound-heterozygous mutations in the *IGHMBP2* gene. The disease began in late childhood and progressed slowly: both sisters were still able to work, drive a car, and walk with the aid of a cane and a silicone ankle–foot orthosis. Although the younger sister had a clinically milder form, both presented with bilateral foot drop, distal weakness, atrophy of the upper and lower extremities, absent reflexes, loss of sensation in feet and hands, no cranial nerve involvement, and a trombone-shaped tongue. Chest X-ray was normal and there were no respiratory problems, meaning that the disease was not diaphragmatic SMA. These findings were consistent with the typical form of CMT2. On the basis of these findings, IGHMBP2-related CMT2 cases are now referred to as CMT2S.

In 2015, Schottmann et al. reported five patients with neuropathy from three families who carried truncating mutations in *IGHMBP2* [89]. In contrast to the findings in SMARD1 patients, they did not manifest respiratory distress, but had progressive sensorimotor neuropathy. Only one patient required nocturnal mask ventilation: the patient’s chest X-ray at the age of 24 years showed bilateral diaphragmatic eventration. However, four others maintained normal respiratory function into childhood and beyond. All patients had a predominantly axonal sensorimotor neuropathy with subsequent muscle atrophy, but without obvious sensory symptoms. Two patients had signs of autonomic neuropathy. These patients were also considered to have been affected by CMT2S.

According to the case studies mentioned above, IGHMBP2-related disorder causes variable phenotypes with respiratory failure: infantile SMARD1 with serious respiratory failure, juvenile SMARD1 with mild respiratory failure, CMT2S with mild respiratory failure, and CMT2S with no respiratory problems. The presence of respiratory failure of varying severity suggests a continuous spectrum of IGHMBP2-related disorders. Infantile SMARD1 and adult CMT2S could be considered as opposite ends of this spectrum.

Here, we briefly discuss arthrogryposis of non-5q-SMA. In 2016, Lingappa et al. reported two SMARD1 infants with arthrogryposis [90]. According to Lingappa et al., the presence of arthrogryposis with early respiratory failure with or without eventration of the diaphragm strongly points to neuromuscular pathology. Arthrogryposis is frequently observed in diseases classified into the non-5q-SMA group. In fact, arthrogryposis has been reported in many diseases discussed in this review article: SMARD1 due to mutations in *IGHMBP2*, XL-SMA due to mutations in *UBA1*, SP-SMA due to mutations in *TVPV4*, SMA-LED1 due to mutations in *DYNC1H1*, and SMA-LED2 due to mutations in *BICD2*. However, the conditions of contracture vary widely: small-to-large numbers of joints may be involved, and mild-to-severe deformities may occur.

### 4.3. Genetics

In 1999, Grohmann et al. reported nine patients from three families with diaphragmatic SMA showing an autosomal recessive mode of inheritance [91]. According to them, diaphragmatic SMA is genetically heterogeneous, and one form is linked to chromosome 11q13–q21. They named the disease associated with the locus that they mapped as SMARD (spinal muscular atrophy with respiratory distress).

Two years later, in 2001, they mapped the gene locus more precisely to chromosome 11q13.2–q13.4, and identified a causative gene, *IGHMBP2* [35]. Here, they used the term “SMARD1”, instead of just “SMARD”. Their report included descriptions of frameshift deletion, in-frame deletion, nonsense, splice donor-site, and recessive missense mutations.

The *IGHMBP2* gene encodes Immunoglobulin Mu (μ) DNA Binding Protein 2 (IGHMBP2), which acts as a helicase for both DNA and RNA [81]. IGHMBP2 includes three domains: helicase domain, R3H (Arg-X-X-X-H) domain, and ZnF (zinc finger) domain. The helicase domain can bind RNA itself and exert ATPase activity, while the R3H domain enhances these functions [81].

Most of the missense mutations in this gene are found in the helicase domain. The location and type of mutations do not appear to correlate with the severity of clinical features [82,83,84,85]. Even so, Cottenie et al. stated that mutations in CMT2 appeared to be less severe than those in SMARD1. In addition, studies of fibroblasts and lymphoblasts indicated that the IGHMBP2 protein levels are significantly higher in CMT2 than in SMARD1, but lower than in controls, suggesting that the differences in clinical phenotype are related to the IGHMBP2 protein levels [84]. Besides the location and type of mutations, we must consider the presence of disease-modifying genes that affect the phenotypic variation in IGHMBP2-related disorders [81].

### 4.4. Molecular Pathogenesis

IGHMBP2 is ubiquitously expressed, but its expression level varies in the central and peripheral nervous systems during development [81]. IGHMBP2 is thought to be involved in many cellular processes, such as pre-mRNA processing, immunoglobulin class switching, the regulation of DNA replication, and interactions with TATA-binding protein [81].

In 2009, Guenther et al. described the ATPase and helicase activity of IGHMBP2 [92]. IGHMBP2 is an ATP-dependent helicase, which unwinds RNA and DNA duplexes in vitro. This helicase localizes predominantly to the cytoplasm of neuronal and non-neuronal cells and associates with ribosomes. SMARD1-causing amino acid substitutions in IGHMBP2 do not affect ribosome binding, yet severely impair ATPase and helicase activity. Guenther et al. proposed that IGHMBP2 is functionally linked to translation, and that mutations in its helicase domain interfere with this function in SMARD1 patients. The schematic in Figure 2 is based on their hypothesis.

In 2009, de Planell-Saguer et al. suggested that IGHMBP2 interacts with ABT1 (protein in humans) [93]. They obtained experimental results showing that a BAC transgene derived from CAST/EiJ mice rescued the motor neuron degeneration of nmd mice (SMARD1 model animal) without restoring the protein levels of IGHMBP2. The BAC transgene contained tRNA genes and the activator of basal transcription 1 gene (*Abt1*). Abt1 (protein in mice) is an essential protein for ribosome biogenesis. As such, de De Planell-Saguer et al. concluded that IGHMBP2 may be associated with ribosome and tRNA, both of which are essential for mRNA translation [93]. Meanwhile, in 2023, Vadla et al. reported that intracerebroventricular injection of self-complementary AAV9 vector with Abt1 (scAAV9-*Abt1*) decreases the disease pathology of FVB-Ighmbp2^nmd/nmd^ (a SMARD1 model animal), significantly increases lifespan, and substantially decreases neuromuscular junction denervation [94]. These findings suggest that IGHMBP2 and ABT1 have a complementary relationship.

In 2024, Prusty et al. pointed out that IGHMBP2 may be associated with the TRanscription and EXport (TREX) complex [95]. TREX complex is a conserved multi-subunit complex essential for embryogenesis, organogenesis, and cellular differentiation throughout life [96]. By linking transcription and mRNA processing and export together, this complex exerts a physiologically vital role in the gene expression pathway [96]. Prusty et al. observed that the absence of IGHMBP2 caused ribosome stalling at the start codon of target mRNAs, leading to reduced translation efficiency for the mRNAs [95].

Disturbance in tRNA production and/or translation to proteins due to a reduction in wild-type IGHMBP2 appears to be critical for the pathogenesis of SMARD1 or CMT2S. However, the mechanisms underlying the selective degeneration of some motor neurons remain unknown. It is unclear which abnormalities contribute most to disease development.

## 5. Spinal Muscular Atrophy, Infantile, James Type (SMA-JI) [Alternative Titles (Symbols): Distal Spinal Muscular Atrophy Type V (dSMAV)/Charcot–Marie–Tooth Type 2D (CMT2D)]

### 5.1. Summary of the Disease

The disease-causing gene of SMAJI, dSMAV, and CMT type 2D, is the *glycosyl-tRNA synthetase* gene (*GARS*), which is mapped to chromosome 7p14 and encodes a glycosyl-tRNA synthetase that plays an important role in protein synthesis.

**Figure 3:**
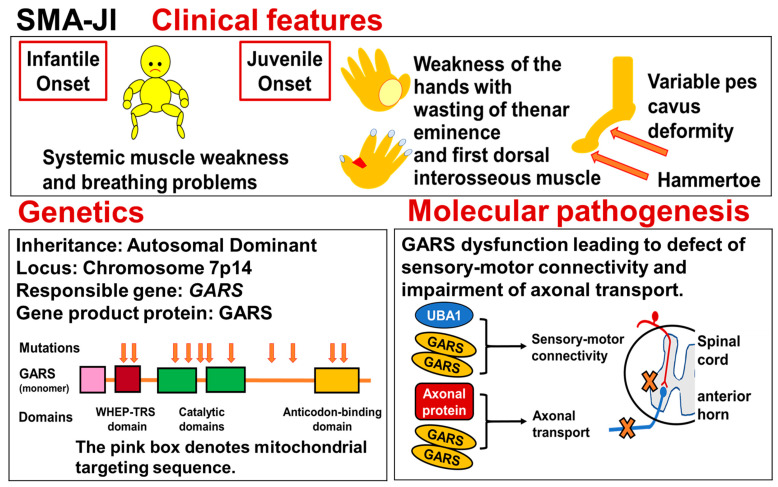
Outline of SMA-JI. Infants with this disease present with respiratory distress, poor feeding, and muscle weakness (distal greater than proximal). Juvenile-onset patients may present with muscle weakness in the hands and legs (as in the typical peroneal muscular atrophy with foot drop and a high steppage gait). *GARS* is a tRNA synthetase gene, which encodes both cytosolic and mitochondrial isoforms of the protein. These forms differ by a 54 amino acid N-terminal mitochondrial targeting sequence. WHEP-TRS domain may play a role in the association of tRNA-synthetases into multienzyme complexes. GARS normally forms a homodimer. *GARS* mutation (or GARS dysfunction) may lead to defects of sensory-motor connectivity and impairment of axonal transport in the spinal cord [97].

Patients with GARS-related neuromuscular disorders have severe weakness and atrophy of the hands and mild-to-moderate weakness of the feet. The disease developed in infancy accompanied by generalized muscle weakness and dyspnea. The mode of inheritance is autosomal dominant. An outline of the disease entity is shown in Figure 3.

### 5.2. Clinical Features

In 1995, Christodoulou et al. reported an autosomal dominant distal form of SMA (or HMN) mainly affecting the upper limbs in a large Bulgarian family [98]. According to the data from 30 patients of this family, the disease onset occurred at a mean age of 17 years (median 16) with selective wasting of the thenar eminence and first dorsal interosseous muscle. Forty percent of the patients subsequently developed symptoms in their feet within about 2 years after onset. One branch of the family has mild pyramidal signs and, rarely, upward plantar responses. There are no sensory symptoms or signs except for slightly reduced vibration sense in the feet of 10% of the patients. The disease was designated as dSMAV by Christodoulou et al. [98], and the causative mutation of this family was discovered in *GARS* [36].

In 1996, Ionasescu et al. reported 26 patients exhibiting peculiar symptoms in a seven-generation family in North America [99]. The presented pedigree showed that 17 men and 9 women were affected in this family. Onset of the disease was between 16 and 30 years of age with weakness of the hands. Patients had severe weakness and atrophy of the hands and mild-to-moderate weakness of the feet. They also showed distal hypesthesia (or an abnormally weak sense) for touch, proprioception, and sensing vibration, which was more evident in the hands than in the feet. Variable pes cavus deformity (an abnormally high plantar longitudinal arch) and/or hammertoes were present in all of the patients. Scoliosis was present in some patients. The disease was designated as CMT2D by Ionasescu et al. [99], and the causative mutation of this family was identified in *GARS* [36].

In 1998, Sambuughin et al. identified a multigenerational Mongolian family with 17 members affected by either dSMAV or CMT2D [100]. The study suggested that dSMAV and CMT2D could occur in the same family. The causative mutation of this family was also found in *GARS* [36].

In 2006, James et al. screened 100 patients with inherited and sporadic lower motor neuron degeneration and identified three novel missense mutations in *GARS* [101]. One mutation, p.Gly652Ala, was in the anticodon binding domain and associated with onset in early childhood and predominant involvement of the lower limbs.

In 2012, Eskuri et al. reported monozygotic twin girls with an onset of weakness in infancy and the same *GARS* mutation, p.Gly652Ala, within the anticodon binding domain [102]. The severity and remarkable similarity in the phenotypes of these girls and the reported case suggest that mutations within the anticodon binding domain are more damaging to aminoacyl tRNA synthetase function than those within other domains of GARS.

In 2020, Markovitz et al. and in 2022, Huang et al. reported severe cases of dSMAV/CMT 2D that developed in infancy and were accompanied by generalized muscle weakness and dyspnea [103,104]. According to Markovitz et al., severe infantile cases with dSMAV also exhibited symptoms of systemic muscle weakness and breathing problems similar to 5q-SMA type 0/type I or SMARD1. Chest X-rays often show evidence of right diaphragm elevation. Muscle pathology shows evidence of neurogenic muscle atrophy. The severe cases with infantile-onset were designated as GARS1 infantile-onset (iSMA) by Markovitz et al. [103].

### 5.3. Genetics

In 1995, Christodoulou et al. mapped the gene locus for dSMAV on chromosome 7p14 [98]. The following year, Ionasescu et al. mapped the gene locus for CMT2D on chromosome 7 [99]. In 2003, Antonellis et al. reported that dSMAV and CMT2D are caused by abnormalities in *GARS* [36]. The gene is located on chromosome 7p14 and encodes Glycyl-tRNA Synthetase (GARS).

*GARS* is one of 37 aminoacyl-tRNA synthetase genes [97]. It encodes both cytosolic and mitochondrial isoforms of the protein, which differ by a 54 amino acid N-terminal mitochondrial targeting sequence [97]. The protein region common to both isoforms has four functional domains: the WHEP-TRS domain, the two domains that form the catalytic core, and an anticodon binding domain (Figure 3). The WHEP-TRS domain is present in a number of higher eukaryote aminoacyl-tRNA synthetases [105], and they may play a role in the association of tRNA-synthetases into multienzyme complexes [106].

The findings of Antonellis et al. strongly suggest that dSMAV and CMT2D belong to the same disease entity [36]. A review of these studies indicates that when seeing a patient with suspected distal SMA/CMT2D, one should determine whether the patient has a family history suggestive of CMT or SMA symptoms. However, it should be noted that sporadic cases without family history have also been reported [101].

Hirayama disease (HD) is known to exhibit upper extremity-predominant symptoms similar to diseases caused by *GARS* mutations. HD usually occurs sporadically, but patients with HD-like conditions should also undergo genetic testing for *GARS*. Although no pathogenic *GARS* mutations were found in the cohort of Blumen et al. [107], they made valuable efforts to diagnose this disease.

### 5.4. Molecular Pathogenesis

In 2010, Motley et al. suggested nine possibilities regarding the molecular pathogenesis of dSMAV/CMT2D [97]: (1) loss of charging function, (2) aggregation, (3) mischarging, (4) nucleolar dysfunction, (5) dimerization, (6) non-canonical functions, (7) disease-associated mutations lead to new, toxic, protein-protein or protein-RNA interactions, (8) mitochondrial toxicity or dysfunction, and (9) impaired axonal transport. Please refer to the original paper for a detailed explanation of each hypothesis. In Figure 3, we present the ninth hypothesis suggesting the impairment of axonal transport.

In 2018, Shorrock et al. reported that aminoacyl-tRNA synthetases (ARSs), including GARS, were identified as downstream targets of UBA1 (ubiquitin-like modifier activating enzyme 1; see the next Section (Section 6) [108]. Regulation of GARS by UBA1 occurred through a non-canonical pathway independent of ubiquitylation. Dysregulation of UBA1/GARS pathways in spinal muscular atrophy mice disrupted sensory neuron fate. However, the sensory neuron defect was corrected following restoration of UBA1 expression and UBA1/GARS pathways in the mice. Based on these findings, Shorrock et al. concluded that defective sensory-motor connectivity in spinal muscular atrophy results from perturbation of the UBA1/GARS pathway [108]. We also present this finding in Figure 3.

Finally, further research is needed to clarify the relationship between genetic abnormalities in ARSs and neurological diseases. Genetic abnormalities in ARSs cause not only dSMA (or HMN)/CMT but also other neurological diseases [109].

## 6. X-Linked Infantile Spinal Muscular Atrophy (XL-SMA) [Alternative Title (Symbol): Spinal Muscular Atrophy, X-Linked 2 (SMAX2)]

### 6.1. Summary of the Disease

**Figure 4:**
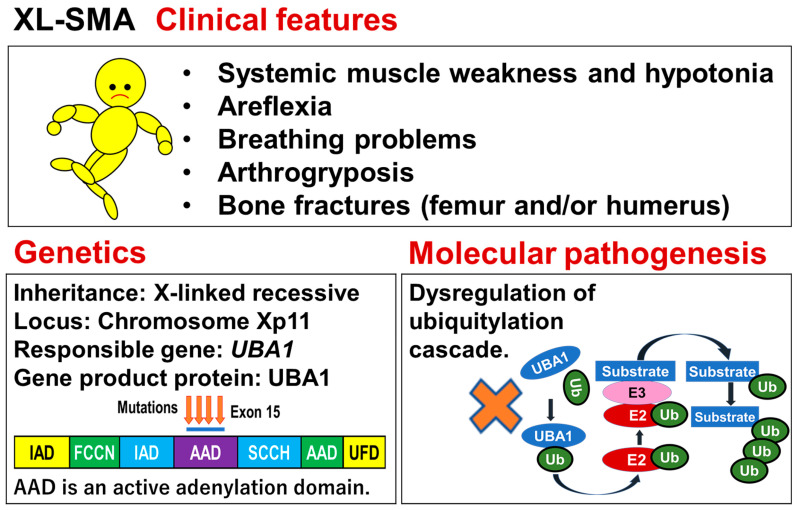
Outline of XL-SMA. The molecular pathogenesis involves the impairment of adenylation and thiolation of ubiquitin (Ub) by UBA1. However, it remains unknown how mutations in the active adenylation domain (AAD) domain (*UBA1* exon 15) cause the disease. Here, the canonical pathway of ubiquitylation is shown, but regulation of GARS by UBA1 occurred through a non-canonical pathway independent of ubiquitylation, as mentioned in Section 5.

X-linked infantile SMA (XL-SMA) or spinal muscular atrophy, X-linked 2 (SMAX2) is a rare but severe form of motor neuron disease, showing hypotonia, areflexia, and arthrogryposis associated with loss of anterior horn cells and death in infancy. Hereinafter, this disease is referred to as XL-SMA. Patients with XL-SMA often present with long bone fractures at birth. This disease is caused by mutations of *UBA1*, which is mapped to chromosome Xp11. An outline of this disease is shown in Figure 4.

Note: Spinal muscular atrophy, X-linked 1 (SMAX1), has been described as spinal and bulbar muscular atrophy [44]. SMAX1 is an adult-onset SMA, and it will not be discussed in this review article.

### 6.2. Clinical Features

In 1988, Greenberg et al. reported four male infants who had hypotonia, areflexia, arthrogryposis (multiple joint contractures) of the fingers, elbows, knees, and hips, and fractures of the femur and/or humerus at birth [110]. These patients were from three sibships in an extended family. Their muscle biopsy findings were consistent with those of SMA type I. Three of the four patients died before 2 years of age. No autopsy was possible in these three infants, and thus there was no direct proof of an anterior horn cell disease. Numerous observations, however, supported a diagnosis of SMA. Together with a pedigree study of this family, Greenberg et al. concluded that this disease represented an X-linked, recessive form of SMA.

Notably, XL-SMA could not be diagnosed without a pedigree study until genetic testing became available. Brandt reported a patient with features very similar to previously reported cases of XL-SMA, but no pedigree for this case was reported [111]. Thus, this case cannot be diagnosed as XL-SMA. Meanwhile, a case reported by Borochowitz et al. was born to consanguineous parents, with no information on whether the mode of inheritance was autosomal recessive or X-linked [112]. Thus, their cases could not be assigned to XL-SMA. However, it is now possible to confirm the diagnosis of XL-SMA by analyzing the causative gene, *UBA1*.

### 6.3. Genetics

In 1995, Kobayashi et al. reported that the XL-SMA locus was mapped to chromosome Xp11.3–q11.2 in the family previously reported by Greenberg et al. [113]. They conducted linkage analysis using multiplex CA repeat markers, meiotic breakpoint mapping (concordance analysis), and whole-X-chromosome multipoint linkage analysis on a family with X-linked arthrogryposis. Later, Dressman et al. narrowed down the disease gene interval to Xp11.3–Xq11.1 by adding new XL-SMA families and new markers [114].

In 2008, Ramser et al. finally identified a mutation causative of this disease in *UBA1*, which is located within the above-mentioned XL-SMA locus (Xp11.3–Xq11.1) [37]. *UBA1* encodes ubiquitin-like modifier activating enzyme 1 (UBA1). Since the discovery of *UBA1* as the gene causative of XL-SMA, Dlamini et al. (2013), Jędrzejowska et al. (2015), Shaughnessy et al. (2020), Wang et al. (2020), and Öztürk et al. (2022) reported mutations of *UBA1* in patients with XL-SMA [115,116,117,118,119].

Strikingly, all of the missense mutations causing XL-SMA are located in exon 15 of *UBA1.* The synonymous C>T substitution leads to an alteration of the methylation pattern of exon 15, resulting in a significant reduction in *UBA1* expression. *UBA1* missense mutations also cause a disease involving overlapping inflammatory-hematological features called VEXAS (vacuoles, E1, X-linked, autoinflammatory, somatic) syndrome. However, the missense mutations causing XL-SMA may not cause VEXAS syndrome [120]. Additionally, it should be noted that VEXAS is a syndrome due to somatic *UBA1* mutations, unlike the XL-SMA due to germline *UBA1* variants.

### 6.4. Molecular Pathogenesis

UBA1 functions at the apex of the enzymatic ubiquitination cascade, which regulates many cellular processes, including the ubiquitin–proteasome system (UPS), transcription, DNA repair pathways, cell cycle progression, translation, metabolism, immune signaling, selective autophagy, vesicle transport, and apoptosis [121].

However, it remains unknown how mutations in *UBA1* exon 15 cause the disease. Although much remains unknown in the molecular pathogenesis of XL-SMA, recent advances in the study of the molecular crosstalk between UBA1 and other SMA-associated proteins are of great interest and will be discussed in the section on SMA-JI (Section 5) and in the Discussion.

## 7. Scapuloperoneal Spinal Muscular Atrophy (SP-SMA) [Alternative Titles (Symbols): Congenital Distal Spinal Muscular Atrophy (CDSMA)/Congenital Spinal Muscular Atrophy and Arthrogryposis (CSMAA)/Hereditary Motor and Sensory Neuropathy Type I1C (HMSN IIC)/Charcot–Marie–Tooth Disease Type 2C (CMT2C)]

### 7.1. Summary of the Disease

**Figure 5:**
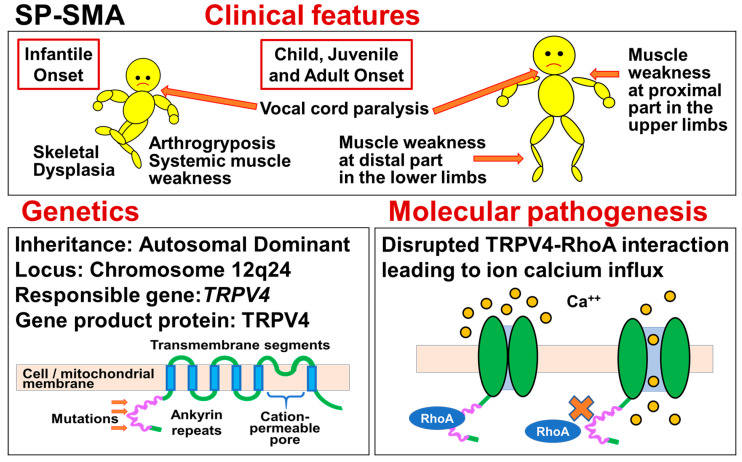
Outline of SP-SMA. Infantile-onset patients show arthrogryposis, systemic muscle weakness, vocal cord paralysis and bone abnormalities. Juvenile- and adult-onset patients present with atrophy of the shoulders, and peroneal and small hand muscles resulting in distal weakness. Causative mutations occur in the ankyrin repeat domain (ARD) of the TRLV4 channel in the cell/mitochondrial membrane. Disrupted TRPV4-RhoA interaction may lead to an abnormal influx of calcium ions.

Scapuloperoneal spinal muscular atrophy (SP-SMA), congenital distal spinal muscular atrophy (CDSMA), congenital spinal muscular atrophy and arthrogryposis (CSMAA), hereditary motor and sensory neuropathy type I1C (HMSN IIC), and Charcot–Marie–Tooth disease type 2C (CMT 2C) are caused by a heterozygous mutation in the *transient receptor potential vanilloid 4* (*TRPV4*) gene located on chromosome 12q24. They are now placed on the spectrum of TRPV4-related neuromuscular diseases. In patients with CDSMA/CSMAA, arthrogryposis and muscle weakness are dominant in infancy. Patients with SP-SMA/HMSN IIC/CMT2C present with atrophy of the shoulders, and peroneal and small hand muscles resulting in distal weakness. Other common features of TRPV4-related neuromuscular diseases include vocal cord paralysis, scoliosis, and/or arthrogryposis. Phenotypic overlap can occur among the diseases. An outline of these diseases is shown in Figure 5.

### 7.2. Clinical Features

The clinical features of SP-SMA, the most representative disease among the TRPV4-related neuromuscular diseases, are muscle weakness and motor dysfunction around the scapula and fibula. Generally, muscle weakness is proximal in the upper limbs and distal in the lower limbs [44]. Other common symptoms include arthrogryposis, vocal cord paralysis, and bone abnormalities.

In the history of clinical research on SP-SMA, attention first focused on arthrogryposis, then vocal cord paralysis, and finally bone abnormalities. Here, we describe these symptoms in this order. As for arthrogryposis, in 1985, Fleury and Hageman reported a lower motor neuron disorder with associated arthrogryposis at birth (CDSMA/CSMAA) in a four-generation family in the Netherlands [122]. The mode of inheritance was autosomal dominant. Overall, 21 of 44 family members examined in the study showed a non-progressive congenital lower motor neuron disorder restricted to the lower extremities. Weakness was most remarkable in the distal parts of the legs. Arthrogryposis was also found in 15 family members. Foot deformity (pes cavus), and lordosis/kyphoscoliosis developed with time in many cases.

As for vocal cord paralysis, in 1992, Delong and Siddique reported a large New England kindred with SP-SMA bearing an autosomal dominant neurogenic amyotrophy [123]. Clinical features included congenital absence of muscles (as quoted from the original paper; the authors probably meant decreased muscle mass), progressive scapuloperoneal atrophy, laryngeal palsy, and progressive distal weakness and atrophy. Among their cases, no instances of sensory disturbance or hearing loss were recorded.

In 1994, Dyck et al. reported two families with HMSN IIC bearing an autosomal dominant syndrome including vocal cord paralysis, respiratory involvement (diaphragm and intercostal muscle), and muscle weakness in hands and legs [124]. In Family1, the most common symptoms were hoarseness and asthma-like stridor. These symptoms were reported in 8 of 11 affected family members. Most of the patients presented with hoarseness that had been present since early in life. Weakness and atrophy of the muscles in the hands and legs were the next most common symptoms. Sensory disturbance was recorded in 7 of 11 affected family members, but no hearing loss was described. Patients with neuromuscular diseases who exhibited vocal cord paralysis have previously been reported. However, it was Dyck’s report that strongly linked this characteristic symptom to neuromuscular disease, leading to the subsequent discovery of the disease-causing gene [38].

In 1999, Donaghy et al. published a second report on HMSN IIC associated with vocal cord paralysis and muscle weakness in the hands and legs. Of note, in the cases of that study, symptoms began in adulthood rather than in infancy or childhood [125].

Next, we describe skeletal dysplasia related to SP-SMA. In the literature, bone deformity-related complications are being reported increasingly frequently. The TRPV4-related neurological disease group has now been characterized by muscle weakness, vocal cord paralysis, and bone abnormalities [126,127,128,129,130]. Skeletal dysplasia associated with SP-SMA includes short stature, brachydactyly, and spondylometaphyseal dysplasia.

In 2014, Echaniz-Laguna et al. described 12 patients with *TRPV4* mutations causing neuropathy (see below), vocal cord paralysis, and/or skeletal dysplasia [128]. All patients presented with skeletal dysplasia, and bone abnormalities were mild and mainly consisted of short limbs at birth, abnormal vertebral ossification, spondylometaphyseal dysplasia, flared metaphyses, mild short stature, retarded carpal ossification, and mild platyspondyly [128]. These results suggest that *TRPV4* mutations may cause impaired endochondral ossification.

In recent years, attention has been drawn to not only bone deformities but also hearing loss as a symptom of SP-SMA [38,127,128,131].

### 7.3. Genetics

In 1996–2005, Genetic linkage analyses mapped the loci of SP-SMA/HMSN IIC/CMT 2C, and CDSMA/CSMAA to chromosome 12q24 [132,133,134].

In 2010, three groups, Landouré et al., Deng et al., and Auer-Grumbach et al., independently reported that heterozygous *TRPV4* mutations cause these neurological diseases, SP-SMA/HMSN IIC/CMT 2C [38,39,40]. *TRPV4* is located on chromosome 12q24 and encodes the transient receptor potential cation channel, vanilloid subfamily, member 4 protein (TRPV4). Landouré et al. examined subjects from two large families with HMSN IIC/CMT 2C [38]. They identified c.C805T and c.G806A in exon 5 of *TRPV4* (p.Arg269Cys and p.Arg269His in the TRPV4 protein). Meanwhile, Deng et al. reported that SP-SMA and HMSN IIC/CMT2C are allelic disorders caused by mutations in *TRPV4* [39]. They found two mutations in two families with SP-SMA and CMT2C: one was c.C946T in exon 6 of *TRPV4* (p.Arg316Cys) in a family with SP-SMA, and the other was c.G806A in exon 5 of *TRPV4* (p.Arg269His) in a family with HMSN IIC/CMT2C. Auer-Grumbach also identified three missense mutations, p.Arg269His, p.Arg315Trp, and p.Arg316Cys, in five families, including patients with CDSMA, SP-SMA, and HMSN2C [40]. Notably, all mutations reported by these three groups were in the ankyrin repeat domain (ARD) of the TRPV4 protein.

CDSMA/CSMAA and SP-SMA/HMSN IIC/CMT 2C have been considered to be autosomal dominant diseases caused by heterozygous mutation. However, a typical infant affected by CDSMA/CSMAA with autosomal recessive inheritance was also reported by Vellila [135]. The infant carried a *TRPV4* mutation (c.281C>T; p.Ser94Leu), but the proband’s parents, who were heterozygous for the mutation, were asymptomatic. However, the authors adopted a cautious approach, and advised the parents to undergo follow-up at a neurology clinic.

### 7.4. Molecular Pathogenesis

Motor and sensory peripheral nerve cells are highly specialized, with long axons that connect the spinal cord with muscle and peripheral sensory organs. These cells depend on ion channels for multiple functions including action potential propagation, synaptic transmission, plasticity, and cell survival [38].

TRPV4 is a well-known cation channel, which is a member of the Transient Receptor Potential (TRP) superfamily [38]. According to Landouré, in *TRPV4*-transfected cells, the CMT2C-causing mutations exerted marked cellular toxicity and increased constitutive and activated channel currents [38]. The remarkable phenotypic variation in TRPV4-associated diseases may be due to changes in the interaction with regulatory proteins in different regions of ARD [38].

In 2014, Takahashi et al. reported that TRPV4 channel activity is modulated by direct interaction of ARD with phosphatidylinositol-4,5-bisphosphate [PI(4,5)P2] [136]. The TRPV4 channel activities were increased by titration or hydrolysis of membrane PI(4,5)P2. Notably, disease-associated *TRPV4* mutations that cause a gain-of-function phenotype abolish PI(4,5)P2 binding and PI(4,5)P2 sensitivity. In 2021, McCray et al. reported that TRPV4 mutations disrupt TRPV4-RhoA interactions and impair neurite extension [137]. Disruption of TRPV4-RhoA interaction may lead to an abnormal influx of calcium [138].

Recently, attention has shifted from TRPV4 in the cell membrane to TRPV4 in the mitochondrial membrane. In 2022, Acharya et al. reported that TRPV4 localizes to a subpopulation of mitochondria in various cell lines [139]. Meanwhile, in 2020, Woolums et al. observed that *TRPV4* mutation disrupts mitochondrial transport and causes axonal degeneration via a CaMKII-dependent elevation of intracellular Ca^2+^ [140].

## 8. Spinal Muscular Atrophy with Progressive Myoclonic Epilepsy (SMA-PME)

### 8.1. Summary of the Disease

**Figure 6:**
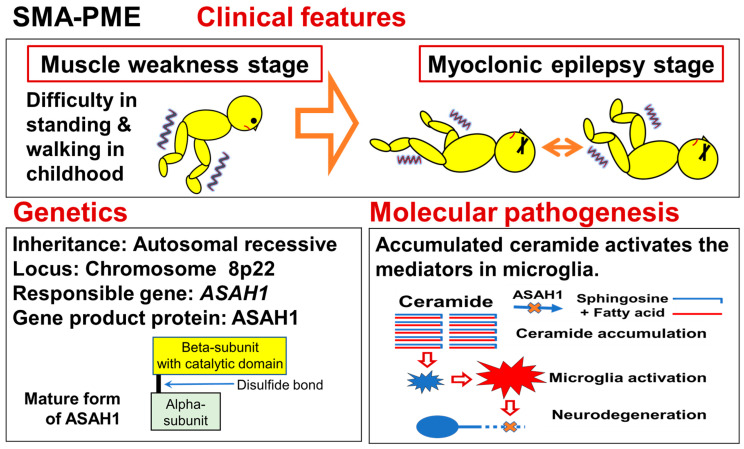
Outline of SMA-PME. Myoclonic epilepsy may occur after muscle weakness appear. Acid ceramidase is a heterodimeric protein composed of a non-glycosylated alpha-subunit and a glycosylated beta-subunit. The beta-subunit of the enzyme catalyzes ceramide to sphingosine and fatty acid. Of the recorded mutations leading to the diagnosis of Farber disease, the majority are located within the beta-subunit. In contrast, a larger number of mutations in SMA-PME have been identified within the alpha-subunit. Accumulation of ceramide may also cause an imbalanced activation of pathways and mediators in microglia, leading to neurodegeneration and neuroinflammation.

SMA-PME is an autosomal recessive neuromuscular disorder characterized by spinal motor neuron dysfunction and myoclonic epilepsy. The disease occurs in infants homozygous for mutations in the *ASAH1* gene, which is mapped to chromosome 8p23.3–p21.3 and encodes N-Acylsphingosine Amidohydrolase 1 (ASAH1). ASAH1 is an acid ceramidase and a key regulator of ceramide metabolism. SMA-PME is progressive, often leading to early death due to respiratory failure. An outline of the disease is shown in Figure 6.

### 8.2. Clinical Features

In 2002, Haliloglu et al. reported four children from two Turkish families affected by a syndrome characterized by severe and progressive myoclonic epilepsy (PME) and proximal weakness, tremor, and lower motor neuron disease, as confirmed by electrophysiological and muscle biopsy findings [141]. All of the children first had difficulty standing and walking and then developed myoclonic epilepsy. Three patients from the same family died in their teens. One patient in their study had generalized spikes and slow waves of 3–3.5 cycles/sec on an electroencephalogram (EEG), and neurogenic findings on muscle biopsy. According to Haliloglu et al., several reports of cases with SMA and myoclonic seizures have been published. Some of the case reports published prior to the paper by Haliloglu et al. did not describe the same disorder, but they did suggest the presence of lower motor neuron diseases associated with myoclonic seizures.

In 2004, Striano et al. reported a patient with child-onset SMA and PME [142]. The patient developed progressive weakness, and slow and clumsy movements in early childhood; his parents noticed gait disturbance, and difficulty in running and getting up from the floor. At the age of 9 years, the patient experienced daily, brief episodes of loss of consciousness, usually accompanied by myoclonic jerks of the upper limbs and, sometimes, with head nodding. EEG showed diffuse slowing in the theta range of background activity with frequent, irregular, generalized spike-and-wave (SW) and polyspike-and-wave (PSW) complexes at 2–2.5 cycles/sec. Muscle biopsy revealed a typical denervation pattern with large groups of small, atrophic type II fibers adjacent to groups of hypertrophied ones.

In 2012, Zhou et al. reported five children from three unrelated families with SMA accompanied by progressive myoclonic epilepsy [41]. The children first developed difficulty walking and then myoclonic seizures. They named lower motor neuron disease with progressive myoclonic epilepsy (SMA-PME). They combined exome sequencing and whole-genome scanning with the use of SNP microarrays to identify the genetic causes of SMA-PME, namely, *ASAH1* mutations, in these families. The results of their genetic study are described below. Following Zhou et al.’s report, case reports of SMA with PME began to be published, including those by Dyment et al. (2014), Filosto et al. (2016), and Radhakrishnan et al. (2021) [143,144,145]. All of the cases described in these reports were diagnosed by observing clinical features and identifying mutations in *ASAH1.*

### 8.3. Genetics

In 2012, Zhou et al. mapped the gene locus for SMA-PME on chromosome 8p22 [41]. Then, they identified *ASAH1* as the disease-causing gene of SMA-PME [41]. The *ASAH1* gene is located on chromosome 8p22 and encodes a lysosomal acid ceramidase, N-acylsphingosine amidohydrolase 1. *ASAH1* is also known as a gene for which mutated forms are responsible for Farber disease [146,147]. Farber disease is a disorder of lipid metabolism that leads to the accumulation of ceramide in tissue. Farber disease is an autosomal-recessive condition resulting from a marked reduction or complete lack of lysosomal acid ceramidase activity [148,149]. Acid ceramidase activity below 10% results in Farber disease, an early-onset disease starting with subcutaneous lipogranuloma, joint pain, and hoarseness [150]. The cause of hoarseness is thought to be ceramides that accumulate around the vocal cords. In 1996, the *ASAH1* gene, which encodes the lysosomal acid ceramidase, was fully sequenced and characterized [151].

Zhou et al. identified a homozygous missense mutation [c.125C>T (p.Thr42Met)] in exon 2 of *ASAH1* in the affected children of two families [41]. The third affected child was compound heterozygous for the same mutation and a deletion of the whole gene. They studied the expression of the c.125C>T mutant cDNA in Farber fibroblasts. The acid ceramidase activity in the transfected fibroblasts was only 32% of that generated by normal cDNA, but this reduced activity could normalize the ceramide level in Farber cells. They also conducted a morpholino-knockdown experiment with the *ASAH1* ortholog in zebrafish, and observed a marked loss of motor-neuron axonal branching, a loss associated with increased apoptosis in the spinal cord. On the basis of the above findings, they hypothesized that a higher residual activity might be responsible for SMA-PME, a later-onset phenotype restricted to the central nervous system (CNS) and starting with lower-motor-neuron disease. Residual levels of acid ceramidase activity may determine the phenotype of the ASAH1-related disorders.

There appears to be a moderate genotype–phenotype correlation in SMA-PME [152]. As shown in Figure 6, acid ceramidase is a heterodimeric protein composed of a non-glycosylated alpha-subunit with a molecular weight (MW) of ~13 kDa and a glycosylated beta-subunit with a MW of ~40 kDa [153]. The beta-subunit of the enzyme catalyzes ceramide to sphingosine and fatty acid. Of the recorded mutations leading to the diagnosis of Farber disease, the majority are located within the beta-subunit. In contrast, a larger number of mutations in SMA-PME have been identified within the alpha-subunit [152].

Recently, Puma et al. described the first case of ASAH1-related pure SMA evolving into Faber disease with multisystemic neurological symptoms in adulthood [154]. The case was compound heterozygous for pathogenic mutations of *ASAH1*, c.124A>G (p.Thr42Ala), and c.413A>T (p.Glu138Val). This case suggests that ASAH1 enzyme activity may change with age.

### 8.4. Molecular Pathogenesis

The exact mechanism by which ASAH1 deficiency-induced ceramide accumulation leads to neuron dysfunction remains unclear. In 2017, Scheiblich et al. reported that ceramide acts as a positive modulator of nucleotide-binding oligomerization domain-like receptor (NOD-like receptor) pyrin domain containing 3 (NLRP3) inflammasome assembly and the resulting release of IL-1β 148 [155]. IL-1β is a pro-inflammatory cytokine released by microglia, which can exacerbate injury when present at elevated levels.

In 2022, Wang et al. indicated that microglia-related signaling and mediators are closely related to the development of motor neuron diseases [80]. They summarized that pathways and mediators are associated with microglia, such as fractalkine signaling, purinergic signaling, nuclear factor-κB (NF-κB) signaling, p38 mitogen-activated protein kinase (MAPK) signaling, triggering receptor expressed on myeloid cells 2-apolipoprotein E (TREM2-APOE) signaling, Rho-associated coiled-coil containing protein kinase (ROCK) signaling, C1q classical component signaling, and ion channel, which are involved in the activation, proliferation, and inflammation of microglia. Taking the findings together, the accumulation of ceramide may also cause imbalanced activation of pathways and mediators in microglia, leading to neurodegeneration and neuroinflammation.

Regarding the potential treatment strategies for Farber disease/SMA-PME, enzyme replacement therapy with recombinant acid ceramidase therapy and gene replacement therapy with *ASAH1* cDNA are currently being investigated [152,156]. Microglia-related molecules can be a treatment target for motor neuron disease, including SMA-PME [80]. As treatments for ASAH1-related disorders are developed, genetic screening for ASAH1 or screening for acid ceramidase activity will likely be recommended [157].

## 9. Spinal Muscular Atrophy Lower Extremity Dominant 1 (SMA-LED1) [Alternative Title (Symbol): Charcot–Marie–Tooth Disease, Type 20 (CMT20)]

### 9.1. Summary of the Disease

Spinal muscular atrophy lower extremity dominant (SMA-LED) is an autosomal dominant, slowly progressive disorder, characterized by proximal muscle weakness and atrophy predominantly affecting the lower limbs, with mild (or absent) upper limb involvement. Some patients show pes cavus.

SMA-LED is now divided into two groups based on the causative genes: SMA-LED1 is caused by heterozygous mutations in the heavy chain of cytoplasmic dynein gene (*DYNC1H1*), while SMA-LED2 is caused by heterozygous mutations in the bicaudal D homolog 2 (*Drosophila*) gene (*BICD2*). *DYNC1H1* is mapped to chromosome 14q32, while *BICD2* is mapped to chromosome 9q22.3.

**Figure 7:**
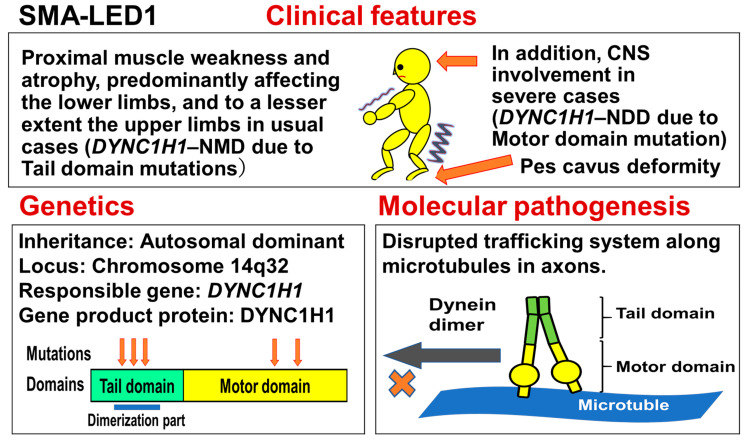
Outline of SMA-LED1. Early reports asserted that causative mutations are located exclusively in the tail domain. However, some causative mutations in the motor domain were later reported. DYNC1H1 is a molecular motor protein required for the retrograde transport of cargo along microtubules in axons and dendrites, and thus is involved in neuronal development, morphology, and survival.

SMA-LED1 caused by *DYNC1H1* mutations may also be classified into two groups based on the presence or absence of CNS involvement. An outline of this disease is shown in Figure 7.

### 9.2. Clinical Features

In 2010, Harms et al. reported 10 patients (6 men and 4 women) exhibiting peculiar symptoms, which were later found to be characteristic of SMA-LED1, in a six-generation family in North America [158]. A pedigree analysis suggested autosomal dominant inheritance. Since the age at onset, pattern of weakness, and disease course were consistent across all individuals, only the proband’s case history was provided in detail in the report. The proband’s mother noted underdevelopment of the child’s leg muscles in infancy. The patient did not walk until 18 months of age and his running was always slow. He could never climb stairs without the assistance of a handrail. However, his leg weakness did not progress even into the fifth decade, when he developed increased leg fatigue and pain. On examination at age 49, neurological abnormalities were limited to lower extremity weakness and atrophy. Symmetric wasting was most prominent in the quadriceps, but involved distal leg muscles as well. Mild pes cavus deformity was present, but there were no contractures. The patient’s gait was waddling with excessive lumbar lordosis. Re-examination 4 years later found no significant decline in measured strength. According to the report of this pedigree, none of the 10 patients examined had arthrogryposis or contractures, but 5 had mild pes cavus.

On the basis of the above-mentioned case studies, the clinical entity of SMA-LED1 (autosomal-dominant, non-progressive type of SMA predominantly affecting the lower legs 1) was established. During the last 10 years, CNS abnormalities associated with SMA-LED1 have been recognized. In 2015, Scoto et al. reported a cohort of 30 cases of SMA-LED1 from 16 families [159]. The clinical severity varied, ranging from generalized arthrogryposis and inability to ambulate to exclusive and mild lower limb weakness. In many individuals with cognitive impairment (exhibited by 9 of the 30 cases) who underwent brain MRI, there was an underlying structural malformation resulting in a polymicrogyric appearance.

### 9.3. Genetics

In 2010, Harms et al. found that the disease locus of SMA-LED1 was present in a 6.1-Mb interval on chromosome 14q32 [158]. Two years later, Harms et al. identified one heterozygous missense mutation, c.1750A>C in exon 8 of *DYNC1H1*, in a patient from the SMA-LED1-affected family also analyzed in the earlier study [42]. The amino acid substitution caused by this missense mutation, p.Ile584Leu, occurred in the tail domain of DYNC1H1. The researchers also found two additional heterozygous tail domain mutations (p.Lys671Glu and p.Tyr970Cys) in other SMA-LED patients, confirming that various mutations in the same domain can cause similar phenotypes.

In 2012, Tsurusaki et al. identified a new mutation, p.His306Arg (c.917A>G), in the tail domain of DYNC1H1 in three patients of a SMA-LED1-affected family [160]. The year before that, Weeden et al. had already identified the same gene mutation in a four-generation family with dominant axonal CMT [161]. The disease reported by Weeden et al. was later classified as Charcot–Marie–Tooth disease, axonal, autosomal dominant, type 20 (CMT20). The clinical features of the patients of Weeden et al. were similar to those of the patients of Harms et al. and Tsurusaki et al. This means that the clinical entity of SMA-LED may be closely related to that of axonal CMT, CMT2.

In early studies, SMA-LED-causative mutations were identified exclusively in the tail domain of *DYNC1H1*. However, many mutations were subsequently identified in its motor domain. In 2012, Willemsen et al. also reported that *DYNC1H1* mutations caused severe intellectual disability and variable neuronal migration defects in families affected by CMT type 2 [162]. Although the term “SMA” was not included in their report, they suggested a relationship between *DYNC1H1* mutations and motor/CNS impairment. Meanwhile, in 2015, Scoto et al. suggested that many *DYNC1H1* mutations in the motor domain were related to malformation of cortical development [159].

In 2020, Becker et al. reported 10 patients with motor and CNS manifestations due to a heterozygous mutation in *DYNC1H1*, suggesting a novel classification of *DYNC1H1*--related disorders: namely *DYNC1H1*-NMD and *DYNC1H1*-NDD [163]. *DYNC1H1*-NMD refers to *DYNC1H1*-related neuromuscular disorders while *DYNC1H1*-NDD refers to *DYNC1H1*-related neurodevelopmental disorders. *DYNC1H1*-NDD presents not only with motor impairment and foot/spine deformities but also with CNS involvement (including cortical malformation).

According to Li et al., most SMA-LED-related mutations in the motor domain were correlated with abnormal brain functions, such as epilepsy and global development delay [164]. All patients carrying *DYNC1H1* mutations in the motor domain presented with deformities and abnormal brain MRI.

### 9.4. Molecular Pathogenesis

The *DYNC1H1* gene encodes the heavy chain of cytoplasmic dynein-1, a motor complex that transports cargo (organelles, vesicles, and macromolecules) toward microtubule minus ends (retrograde transport) in axons and dendrites [165].

Crudely, mutations in the tail domain (close to the N-terminus) usually cause pure motor neuron defects, whereas mutations in the motor domain (close to the C-terminus) cause abnormalities in cortical development. Notably, Li et al. reported that all patients with mutations in the motor domain of *DYNC1H1* presented with deformities and abnormal brain MRI [164].

Mutations in the tail domain can shorten the run length of the dynein–dynactin–BICD2 complex along microtubules, whereas mutations in the motor domain can disrupt the interaction between dynein and microtubules [165]. According to Becker et al., the predominant involvement of the lower extremities in many cases of SMA-LED1 (DYNC1H1-NMD) is due to the longer neuronal transportation distance compared with that in the upper extremities and cortex [163]. Such an explanation seems consistent with a mechanism of “length-dependent weakness” in distal HMNs [32].

## 10. Spinal Muscular Atrophy Lower Extremity Dominant 2 (SMA-LED2) [Alternative Title (Symbol): Distal Congenital Spinal Muscular Atrophy (DCSMA)]

### 10.1. Summary of the Disease

Spinal muscular atrophy lower extremity dominant 2 (SMA-LED2)/distal congenital spinal muscular atrophy (DCSMA) is an autosomal-dominant SMA, presenting with congenital slowly progressive muscle weakness of the lower limbs (the upper limbs are also sometimes involved) and bone deformity. SMA-LED2 is caused by heterozygous mutations in the bicaudal D homolog 2 (*Drosophila*) gene (*BICD2*), which is mapped to chromosome 9q22.3.

**Figure 8:**
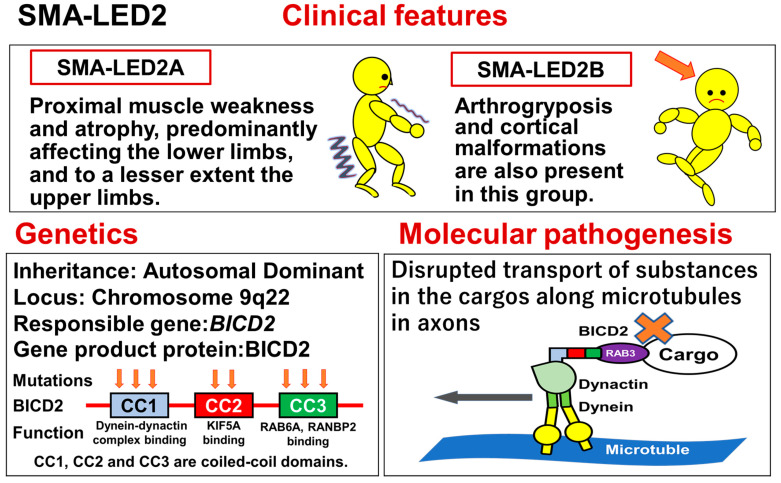
Outline of SMA-LED2. Cytoplasmic dynein is a molecular motor protein required for the retrograde transport of cargo along microtubules in axons and dendrites, as described in the previous Section (Section 9). The BICD2 protein is an adaptor protein for linking the cargo to the motor protein.

However, SMA-LED2 (also known as BICD2-opathies) has been divided into two forms based on the disease severity: SMA-LED2A (mild) and SMA-LED2B (severe). Both of these forms are caused by heterozygous mutations of the bicaudal D homolog 2 (*Drosophila*) gene (*BICD2*). An outline of these diseases is shown in Figure 8.

### 10.2. Clinical Features

In 1994, Frijns et al. reported seven patients exhibiting peculiar symptoms, which were later found to be characteristic of SMA-LED2, in a three-generation family in the Netherlands [166]. The patients had contractures in ankles/feet and atrophy and paresis in distal leg muscles. They all had difficulty walking to varying degrees: some of them presented with waddling gait, walking on toes, or had orthopedic casts. However, according to the authors, the phenotype in this family was clearly different from distal arthrogryposis because the hands were not involved. Later, the causal gene in this family was identified as *BICD2* by Neveling et al. [43].

In 2013, Oates et al. reported six kindreds of DCSMA or hereditary spastic paraplegia (HSP). In these kindreds, affected individuals had a mutation in *BICD2* [45]. According to the authors, DCSMA is a disorder of developing anterior horn cells and shows lower-limb predominance and clinical overlap with upper motor neuron involvement. The DCSMA-affected individuals presented with congenital or early-onset hip dislocation, lower-limb contractures, foot deformities, and predominantly distal leg wasting. Weakness was often more marked proximally in the lower limbs; upper-limb involvement was mild. Upper motor neuron (UMN) signs, including brisk reflexes and extensor plantar responses, were present in childhood in severely affected individuals.

In summary, SMA-LED2 (or DCSMA) involves lower limbs and additionally upper limbs in some cases. The age of disease onset varies from congenital to adulthood, but the disease progression is very slow [167]. However, the form mentioned above is now recognized as the classic form of SMA-LED2 or SMA-LED2A [168]. This renaming was undertaken because of successive articles published in 2016–2018 on a new form with arthrogryposis and cortical malformations [169,170,171]. This severe phenotype has been named SMA-LED2B. [168].

### 10.3. Genetics

In 2013, three groups, Neveling et al., Peeters et al., and Oates et al., mapped a gene mutation that causes SMA-LED2A to chromosome 9q22.3 [43,44,45]. The gene identified in this locus is the bicaudal D homolog 2 (*Drosophila*) gene (*BICD2*) encoding the BICD2 protein. Notably, all three groups identified the same mutation, c.320C>T (p.Ser107Leu), in several families with different ethnicities, with one proven de novo occurrence.

Later, in 2016–2018, Ravencroft et al., Storbeck et al., and Koboldt et al. identified *BICD2* mutations in patients with SMA-LED2B [169,170,171], after which focus was placed on the genotype–phenotype correlations. Koboldt et al. analyzed 99 *BICD2* variant carriers from 35 families, suggesting that the variant location in *BICD2* correlates with some clinical features [168]. They found certain apparent trends, but most did not reach statistical significance (*p* < 0.05). For example, individuals with a variant in CC1 were more likely than those with CC2/CC3 variants to have foot deformities (75% vs. 45%, *p* = 0.1467), but less likely to exhibit severe phenotypes including arthrogryposis multiplex congenita (23% vs. 44%, *p* = 0.2919), brain abnormalities (14% vs. 32%, *p* = 0.4203), and respiratory issues (17% vs. 35%, *p* = 0.4083). Note that the CC1, CC2, and CC3 domains of *BICD2* are shown in Figure 4.

### 10.4. Molecular Pathogenesis

*BICD2* is a human ortholog of the *Bicaudal-D* gene *(BicD)* discovered in *Drosophila*. The mechanisms that establish anterior–posterior polarity in insect embryos have been investigated for over a century [172]. *BicD* was found to be one of the key factors determining this polarity. The first-instar larvae of *Drosophila* normally provide a simple, constant pattern of 12 segments visible externally, with characteristic structure and polarity [173]. However, *BicD* mutation was found to perturb the patterning of the larval segments, resulting in a bicaudal (two-tailed) pattern. The anterior region of the first-instar larvae was replaced with the posterior region, consequently doubling the posterior region in the mutant larvae [174].

Human *BICD2* encodes the Bicaudal D2 (BICD2) protein, a golgin and motor-adaptor protein involved in Golgi dynamics and vesicular and mRNA transport [43]. Golgins are a class of Golgi membrane-associated molecules that are involved in the organization of the Golgi apparatus and interact with components of the trafficking machinery [175]. BICD2 interacts with the dynein–dynactin motor complex and with the small GTPase RAB6A to facilitate the trafficking of key cellular cargo, including mRNA, Golgi, and secretory vesicles. All of these are critical to motor neuron development and/or maintenance [45].

Rossor et al. hypothesized that SMA-LED2 muscle exhibits neurotrophin trafficking defects [176,177]. According to their theory, in SMA-LED2, the trafficking defects of muscle-derived neurotrophins to the cell membrane may lead to excessive programmed cell death because the motor neurons would not receive enough secreted neurotrophins to maintain their survival.

Looking at the interactions of BICD2 with other molecules, it is easy to understand that BICD2 functions as a motor adaptor protein. However, the actual role of BICD2 appears to be much more complex because it has been reported to control microtubule stability at the intracellular level and neuronal migration at the brain tissue level.

In 2018, Martinez-Carrera et al. reported that fibroblasts derived from patients with SMA-LED2 exhibited stable microtubules [178]. They asserted that overexpression of mutated *BICD2* increased microtubule stability in motor neurons, which was associated with axonal aberrations such as collateral branching and overgrowth.

Martinez-Carrera et al. also analyzed a *Drosophila* model of SMA-LED2; neuron-specific expression of mutated *BICD2* reduced the NMJ size in larvae and impaired the locomotion of adult flies [178]. The reduction in NMJ size due to mutated *BICD2* suggests that BICD2 regulates not only retrograde but also anterograde transport of cargo.

The role of BICD2 in neuron migration also requires further investigation because aberrant migration of neurons can cause the cortical malformations commonly seen in SMA-LED2B. In 2019, Will et al. observed that BICD2 controls the radial migration of upper-layer cortical neurons in vivo [179]. Their findings may help to explain the CNS abnormalities in SMA-LED2B.

## 11. Discussion

### 11.1. Motor Neuron Impairment of 5q-SMA and Non-5q-SMA

In this review, we present an overview of 5q-SMA and representative non-5q-SMA diseases. Historically, until 1990, 5q-SMA and non-5q-SMA were diagnosed based on symptoms such as muscle weakness and arthrogryposis (multiple joint contractures), along with histological findings of spinal anterior horn cells, motor neurons, and sensory nerves [61]. Since the discovery of the gene causative of 5q-SMA in 1995 [2], emphasis has been placed on identifying disease loci on chromosomes and the molecular genetic diagnosis of non-5q-SMA [33]. Since the diagnosis was established at the DNA level, the molecular pathogenesis of each disease has gradually become clearer, leading to the understanding that 5q-SMA and non-5q-SMA mainly involve failures of critical life-sustaining systems [1].

The role of SMN, which is produced from the 5q-SMA gene (or the *SMN1* gene), remains unclear. SMN may be associated with biosynthesis of snRNPs, axonal transport, and maturation of NMJ, as mentioned in Section 3. In contrast, as described in the previous sections, the central roles of proteins generated from disease-causing genes associated with non-5q-SMA are all quite distinctive. Even so, the molecular pathogenesis that underlies each of the non-5q-SMA diseases remains unclear.

Whether the molecular pathophysiology of 5q- and non-5q-SMA can cross over has also been discussed. In 2014, Wishart et al. reported that SMN physically interacted with UBA1 in neurons, and disruption of *Uba1* mRNA splicing was observed in the spinal cords of SMA mice exhibiting the disease symptoms [180]. Dysregulation of UBA1 and subsequent ubiquitination pathways led to β-catenin accumulation, which promotes motor neuron pathology in vivo [180].

More recently, Šoltić and Fuller suggested the presence of molecular crosstalk between 5q-SMA- and non-5q-SMA-related proteins [181]. According to them, the perturbation of SMN, UBA1, GARS, and lamin A/C may help explain mechanisms of tissue-specific pathology in SMA. They also proposed that Wnt/β-catenin signaling is a common molecular pathway on which each of the SMA-causing gene products converges.

### 11.2. Boundary between Non-5q-SMA and CMT2

The axonal form of CMT (CMT2), causes progressive axonal degeneration and cell death, leading to muscle weakness and muscle atrophy. With advances in DNA analysis, the boundary between non-5q-SMA (or HMN) and CMT2 has become rather blurred. It has recently been revealed that non-5q-SMA diseases overlap with axonal forms of CMT2 [182].

For example, SMA-LED1 with p.His306Arg in DYNC1H1 (c.917A>G in *DYNC1H1*) was found to be a disease similar to that diagnosed as CMT20 with this same mutation [160,161]. Tsurusaki et al. and Weedon et al. independently reported their own patients with similar symptoms, and independently identified the same mutation in *DYNC1H1*. Given this background, SMA-LED1 and CMT20 should be considered to be exactly the same disease.

The next example involves an *IGHMBP2* mutation. *IGHMBP2* is the gene causative of SMARD1, characterized by diaphragmatic palsy in infancy [35]. Patients with SMARD1 present with distal muscle weakness and muscle atrophy. Mutations in the same gene also cause an axonal form of CMT, CMT2S, which presents with slowly progressive distal muscle weakness and muscle atrophy. Patients with CMT2S show no significant respiratory compromise [183]. The phenotypic difference between SMARD1 and CMT2S may be explained by them occupying opposite ends of the phenotypic spectrum of IGHMBP2 disease.

On the basis of current knowledge, non-5q-SMA and CMT2 appear to belong to the same category of neuronal diseases.

### 11.3. Neuropathological Studies of Spinal Cord in Non-5q-SMA Patients

There has been little opportunity to study the neuropathological basis of non-5q-SMA. Here, we will discuss the neuropathological findings of non-5q-SMA patients.

Harms et al. reported a pedigree of SMA-LED1 caused by a *DYNC1H1* mutation [158]. According to their report, the EMG findings and myopathology were consistent with a chronic neurogenic etiology, but could not distinguish between loss of anterior horn cells and degeneration of motor axons. They may not have had an opportunity to examine autopsy samples from a patient with SMA-LED1.

Meanwhile, Deng et al. reported a pathological study with autopsy samples from a patient with SP-SMA caused by heterozygous *TRLV4* mutation [39]. As for the muscle pathology, severe muscle fiber-type grouping and atrophy with variability in fiber size were identified. Both type 1 and type 2 fibers showed atrophy as demonstrated by ATPase staining. These findings were similar to the neurogenic pattern of 5q-SMA. As for the neuropathology in the CNS, normal numbers of motor neurons were observed in the spinal cord of the patient. There was no gliosis in the anterior horns. These findings were not consistent with those of CNS diseases including amyotrophic lateral sclerosis (ALS) and 5q-SMA [184]. This may suggest that peripheral motor neuropathy is the main pathogenesis in SP-SMA. However, it is too early to conclude that neurodegenerative changes in the motor neuron in the spinal cord could be absent even when the disease progresses.

Oates et al. reported completely different pathological data with autopsy samples from a patient with congenital distal spinal muscular atrophy, who did not carry a *TRLV4* mutation [185]. The patient died at 14 months of age from an unrelated illness. There was a reduction in anterior horn cell number in the lumbar region and, to a lesser degree, the cervical spinal cord, and atrophy of the ventral nerve roots at these levels, in the absence of additional peripheral nerve pathology or abnormalities elsewhere along the neuronal axis. Despite the young age of the child at the time of autopsy, there was pathological evidence of ongoing loss or degeneration of anterior horn cells, suggesting that anterior horn cell loss in dominant congenital spinal muscular atrophy occurs early in life, and is largely complete by the end of infancy. These findings confirm that the disease of the patient is a true form of SMA caused by the loss of anterior horn cells localized to the lumbar and cervical regions early in development.

Considering all of the above, motor neuropathy is present in each disease classified into the non-5q-SMA group. However, it is difficult to comment on whether anterior horn cells are involved in the development of such diseases. If motor neuron damage is mild and/or limited to the periphery, anterior horn cells of the spinal cord will not be lost.

### 11.4. Need for Genetic Analysis in the Diagnosis of Non-5q-SMA

Non-5q-SMA diseases have been clinically diagnosed based on the phenotype including specific symptoms, age of onset, pattern of muscle involvement, and mode of inheritance. However, strictly speaking, there are no peculiar symptoms that are specific to this disease group. The presence of some peculiar symptoms cannot alone be grounds for diagnosing the disease, while an absence of peculiar symptoms is not a basis for ruling out the disease.

Here, we will consider a patient with muscle weakness and atrophy accompanied by vocal cord paralysis. Vocal cord paralysis has been considered to be a key clinical aspect for the diagnosis of SP-SMA due to *TRPV4* mutation. However, vocal cord paralysis has also been reported in patients with distal HMN due to *SLC5A7* mutation [186] and SMA-LED2 due to BICD2 mutation [187]. In addition, according to a literature review by Chen et al., 23% of patients with *TRPV4* mutation lack vocal cord paralysis [131].

To reiterate what was stated in Section 2, clinical features cannot distinguish the causative gene. According to the data of Fernández-Eulate et al., *TRPV4* mutations were found only in 3 out of 15 patients with SP-SMA, although it is widely known that the causative gene for SP-SMA is *TRPV4* [8]. *BICD2* mutations were also found in 3 out of 15 patients with SP-SMA, although it is widely known that *BICD2* mutations cause SMA-LED2 [8]. For the diagnosis of patients with non-5q-SMA, it is essential to perform DNA analysis using new technologies based on NGS such as WES and WGS.

### 11.5. Future Prospects for 5q-SMA Therapies

Three drugs are currently available for 5q-SMA: nusinersen, onasemnogene abeparvovec, and risdiplam [3]. According to the survey conducted by Schwab, patients with 5q-SMA and their parents view prenatal testing and therapies positively [188]. To date, two experimental studies on the fetal treatment of 5q-SMA have been reported: gene therapy and *SMN2* splicing modifier therapy [189,190]. The latter approach may be more feasible. Kong et al. reported that intraperitoneal injection with a small molecule *SMN2* splice modifier (SMN-C3) to the pregnant dams restored axonal growth and associated maturation, and prevented subsequent neonatal axon degeneration in the 5q-SMA model mice [190]. Translating the results to humans, pregnant women would take risdiplam, an oral 5q-SMA drug, for their affected babies. However, Kong et al. were careful to state that “although our therapeutic intervention studies in SMA mice show improved outcomes with treatment initiated in utero, our present ability to predict outcomes of SMA fetuses based on present genetic investigations is at best rudimentary” [190].

Entirely new treatment strategies for 5q-SMA have also been devised, but have not yet been approved for clinical use. One possible strategy for SMA treatment is the inhibition of catenin signaling, a downstream system under the control of the causative gene of SMA [180]. It has already been reported that pharmacological inhibition of β-catenin strongly improved neuromuscular pathology in SMA models in zebrafish, fruit flies (*Drosophila)*, and mice. Another possible strategy is targeting the pathways or key mediators associated with microglia [79,80]. Inhibition of microglial activation may ameliorate the symptoms or delay disease progression.

### 11.6. Future Prospects for Non-5q-SMA Therapies

Many genes causative of non-5q-SMA have already been identified, and a substantial body of knowledge about gene variants and their associated phenotypes has been accumulated and is available for elucidating molecular pathogenesis. In the case of 5q-SMA, a comparison between mild and severe cases led to the identification of a modifying gene, *SMN2*, paving the way for the development of treatments aimed at correcting *SMN2* splicing [50]. In non-5q-SMA, as well as 5q-SMA, thorough comparisons between mild and severe cases of the same disease must be performed to search for factors that differentiate them. Unfortunately, at present, disease-modifying therapies are not available for non-5q-SMA.

Toward a gene therapy approach for SMARD1, in 2015, Nizard et al. reported rescue of the disease phenotype in a SMARD1 mouse model after therapeutic delivery by the systemic injection of a self-complementary AAV9 construct encoding wild-type *IGHMBP2* to replace the defective gene [191]. Administration of single-stranded AAV9-*IGHBP2* restored protein levels, motor function, neuromuscular physiology, and longevity (450% increase). To test this strategy in a human model, wild-type IGHMBP2 was introduced into motor neurons derived from human SMARD1-induced pluripotent stem cells. These cells showed increased viability and axonal length in long-term culture. Meanwhile, in 2016 and 2022, Shababi et al. and Smith et al. also reported the successful rescue of SMARD1 model mice with single-stranded AAV9-*IGHMBP2* [192,193]. Their reports suggested the possibility of initiating human clinical trials for gene therapy of SMARD1.

## 12. Conclusions

In this review, we described 5q-SMA and non-5q-SMA diseases. Long before *SMN1* was discovered as a gene causative of 5q-SMA, the existence of non-5q-SMA had already been recognized. However, given that nosological entities should be based on symptomatology, it has been very difficult to properly classify non-5q-SMA. Similar symptoms may not be indicative of a single specific disease, and many different disorders with similar symptoms may exist. To achieve an accurate diagnosis, DNA analysis of the index patient is essential.

To date, many causative genes and their abnormalities have been identified. DNA analysis technologies based on NGS such as WES and WGS are now progressing rapidly, enabling us to identify disease-causing mutations. Once a disease-causing mutation has been identified by DNA analysis, the phenotypic spectrum and molecular pathogenesis of the disease should soon be clarified. Identification of the causative genes enables the development of gene replacement therapy, and clarification of the molecular pathogenesis accelerates the establishment of disease-modifying therapies. We are optimistic that knowledge about the gene abnormalities, phenotypic spectrum, and molecular pathogenesis of the disease will open up the possibility of therapies for non-5q-SMA.

## Figures and Tables

**Table 1:** Comparison between 5q-SMA and non-5q-SMA.

	5q-SMA	Non-5q-SMA
**Similarities**		
Mobility impairment		
Gross motor difficulties	+	+
Fine motor difficulties	+	+
Complications		
Arthrogryposis	+	+
Respiratory failure	+	+
**Frequency in SMA-suspected patients**	50%	50%
		(including undiagnosed cases)
**Differences**		
Incidence in the general population	High	Low
		(each of non-5q-SMA diseases)
Genetic diagnosis	Regular testing with PCR	NGS, WES, WGS
Established Therapies	+	**-**

**Table 2:** Terminology.

Category	Subcategory	Abbreviation
Spinal muscular atrophy (SMA)	SMN1-related SMA	5q-SMA
	Non-SMN1-related SMA	Non-5q-SMA
Charcot—Marie—Tooth disease (CMT)	Demyelinating CMT	CMT1
	Axonal CMT	CMT2
Hereditary neuropathy	Hereditary motor and sensory neuropathy	HMSN
	Hereditary motor neuropathy	HMN
	Hereditary sensory neuropathy	HSN
	Hereditary sensory and autonomic neuropathy	HSAN

**Table 3:** Pediatric SMA diseases covered in this review article.

Disease	Descriptive Name	Muscle Involvement	Inheritance	Locus	Gene	Year *	Section Number
**5q-SMA**							
5q-SMA Type 0	Severest form of 5q-SMA	proximal	AR	5q13	*SMN1*	1995	Section 3
5q-SMA Type I	Severe form of 5q-SMA	proximal	AR	5q13	*SMN1*	1995	Section 3
5q-SMA Type II	Intermediate form of 5q-SMA	proximal	AR	5q13	*SMN1*	1995	Section 3
5q-SMA Type III	Mild form of 5q-SMA	proximal	AR	5q13	*SMN1*	1995	Section 3
5q-SMA Type IV	Mildest form of 5q-SMA	proximal	AR	5q13	*SMN1*	1995	Section 3
**Non-5q-SMA**							
SMARD1	SMA with respiratory distress type 1	proximal and distal	AR	11q13	*IGHMBP2*	2001	Section 4
SMA-JI	SMA, infantile, James type	proximal and distal	AD	7p14	*GARS1*	2003	Section 5
XL-SMA	X-linked infantile SMA	proximal	XR	Xp11	*UBA1*	2008	Section 6
SP-SMA	Scapuloperoneal SMA	proximal and distal	AD	12q24	*TRPV4*	2010	Section 7
SMA-like disorder due to mitochondorial dysfunction	SMA-like presentation with stridor and respiratory insufficiency	proximal and distal	AR	22q13	*SCO2*	2010	No
SMA-PME	SMA with progressive myoclonic epilepsy	proximal and distal	AR	8p22	*ASAH1*	2012	Section 8
SMA-LED1	SMA lower extremity dominant 1	proximal	AD	14q32	*DYNC1H1*	2012	Section 9
SMA-LED2	SMA lower extremity dominant 2	proximal	AD	9q22	*BICD2*	2013	Section 10
SMARD2	SMA with respiratory distress type 2	proximal and distal	AR	Xq12	*LAS1L*	2014	No
SMABF1	SMA with congenital bone fractures-1	proximal and distal	AR	15q22	*TRIP4*	2016	No
SMABF2	SMA with congenital bone fractures-2	proximal and distal	AR	10q22	*ASCC1*	2016	No
SMA-like disorder due to cytoskeletal defects	Neurodevelopmental disorder with hypotonia, neuropathy, and deafness (NEDHND) and respiratory insufficiency	proximal and distal	AR	19q13	*SPTBN4*	2021	No

Alternative disease names are also used to refer to particular diseases, but in this table, a single representative name that includes the term SMA is used for each disease. * The year the gene that causes SMA was confirmed. AR: autosomal recessive inheritance, AD: autosomal dominant inheritance, XR: X-linked recessive inheritance, Year: the year when the gene that causes an SMA form was confirmed.

## Data Availability

No new data were created or analyzed in this study. Data sharing is not applicable to this article.
